# Phosphorylation of MIF by PIP4K2a is necessary for cilia biogenesis

**DOI:** 10.1038/s41419-023-06323-9

**Published:** 2023-12-05

**Authors:** Lu Zhang, Hongbing Zhang, Ewud Agborbesong, Julie Xia Zhou, Xiaogang Li

**Affiliations:** 1 Department of Internal Medicine, Mayo Clinic, Rochester, MN 55905 USA; 2 Department of Nephrology, Renmin Hospital of Wuhan University, Wuhan, 430060 China; 3 Department of Biochemistry and Molecular Biology, Mayo Clinic, Rochester, MN 55905 USA

**Keywords:** Cell biology, Kidney diseases

## Abstract

Primary cilia are microtubule-based organelles that play important roles in development and tissue homeostasis. Macrophage migration inhibitory factor (MIF) has long been recognized as a secreted cytokine in the pathogenesis of various human diseases, including cancer and autosomal dominant polycystic kidney disease (ADPKD). Unlike other cytokines, unique functional characteristics of intracellular MIF have emerged. In this study, we show that MIF is localized and formed a ring like structure at the proximal end of centrioles, where it regulates cilia biogenesis through affecting 1) the recruitment of TTBK2 to basal body and the removal of CP110 from mother centriole, 2) the accumulation of CEP290 at centriolar satellites, and 3) the trafficking of intraflagellar transport (IFT) related proteins. We also show that MIF functions as a novel transcriptional factor to regulate the expression of genes related to ciliogenesis via binding on the promotors of those genes. MIF also binds chromatin and regulates transcription of genes involved in diverse homeostatic signaling pathways. We identify phosphatidylinositol-5-phosphate 4-kinase type 2 alpha (PIP4K2a) as an upstream regulator of MIF, which interacts with and phosphorylates MIF at S91 to increase its interaction with 14-3-3ζ, resulting in its nuclear translocation and transcription regulation. This study suggests that MIF is a key player in cilia biogenesis and a novel transcriptional regulator in homeostasis, which forward our understanding of how MIF is able to carry out several nonoverlapping functions.

## Introduction

Primary cilium is a non-motile sensory and signaling organelle, which is present in diverse cell types in human body [1]. Primary cilium is initiated in a specific region of the plasma membrane, the entire cilia structure is enclosed by the cell plasma membrane, while inside cilia is a microtubule-based cytoskeleton structure called the axoneme which is grown from the basal body [2, 3]. Primary cilia function as cellular antennae on eukaryotic cells to sense specific signaling cues from the extracellular environment, including mechanosensation, chemosensation and thermosensation [4]. Genetic diseases where the assembly and function of primary cilia are defective, ciliopathies, indicate that primary cilia are tightly associated with normal development in mammals [5].

Macrophage migration inhibitory factor (MIF) is one of the first described cytokines, being as a soluble immune cell-derived factor when it has been identified over 50 years ago. MIF is constitutively and ubiquitously expressed in both immune and non-immune cells, such as monocytes/macrophages, B- and T-cells, and endothelial and epithelial cells, in response to stress caused by different factors, leading to pathological conditions [6, 7]. As an inflammatory cytokine, MIF is involved in the carcinogenesis of many cancer types and autosomal dominant polycystic kidney disease (ADPKD) [8–10]. The functions of MIF have exceeded what is implied by its historical name, including that MIF functions as: 1) a tautomerase/isomerase which targets a number of substrates, including D-dopachrome, 2-hydroxyphenylpyruvate (HPP), and 3,4-dihydroxyphenylaminechrome [11], in which this enzymatic activity is required for optimal signaling in inflammatory and tumor growth pathways [6, 12], 2) a poly(ADP-ribose) (PAR) polymerase-1 (PARP-1)–dependent apoptosis-inducing factor (AIF)-associated nuclease (PAAN) [13] to cleave genomic DNA into large fragments, in which its recruitment to the nucleus through an interaction with AIF, and 3) a chaperone protein and a thiol-protein oxidoreductase [14, 15], in which endogenous MIF can be a chaperone of soluble misfolded superoxide dismutase (SOD1) in the cytosol to directly inhibit the accumulation of misfolded SOD1 and its binding to intracellular membranes in neuronal cultures [14]. In addition, MIF can bind with Jab1, a coactivator of AP-1, in the cytosol to inhibit Jab1 and AP-1 transcriptional activity [10]. Targeting MIF decreased Rb-E2F, NF-κB and Ap-1-mediated transcription and increased p53 transcriptional activity, leading to differential expression of cell cycle regulators and subsequent cell cycle arrest in G0/G1 phase [16]. However, whether intracellular MIF is involved in cilia biogenesis has not yet been elucidated.

In this study, we show that MIF is a structure protein of basal bodies, which forms a ring-like structure at proximal end of centrioles to regulate cilia assembly and elongation through defined ciliary proteins, including CP110, TTBK2, CEP290, and IFT related proteins, The phosphatidylinositol-5-phosphate 4-kinase type 2 alpha (PIP4K2a) is an upstream regulator of MIF, which interacts and phosphorylates MIF at S91 to increase the interaction of MIF with 14-3-3ζ, resulting in MIF nuclear translocation. Nuclear MIF functions to regulate the transcription of ciliary related genes.

## Results

### Intracellular MIF is dynamically associated with cilia assembly and disassembly during cell cycle

**Fig. 1:**
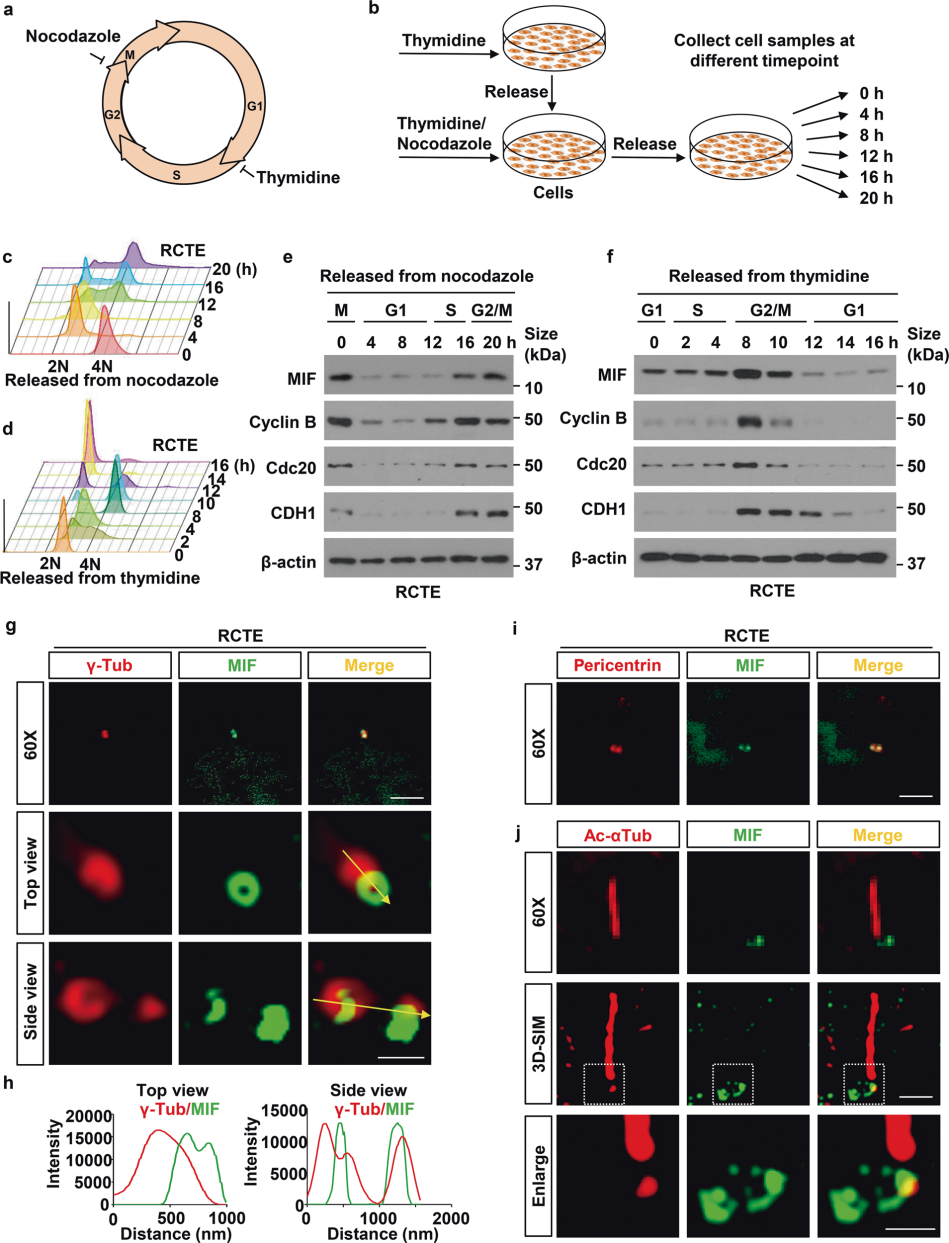
The levels of MIF protein fluctuate throughout cell cycle and MIF is located at basal body in RCTE cells. **a** Schematic showing the cell cycle phase inhibited by nocodazole and thymidine. **b** Experimental outlines for using thymidine-nocodazole block or double thymidine block methods to synchronize RCTE cells. Fluorescence-activated cell sorting (FACS) was used to estimate the cell-cycle profiles in thymidine/nocodazole (**c**) and double thymidine block (**d**) treated cells by measuring the DNA contents in those cells with propidium iodide staining. Western blot analysis of whole cell lysates derived from RCTE cells synchronized in M phase by thymidine-nocodazole (**e**) and in late G1/S phase by double thymidine treatment (**f**) following by releasing back into the cell cycle. **g** Representative images of RCTE cells stained with MIF antibody and co-stained with γ-tubulin (γ-Tub) (red), which were visualized under 2D microscope and three-dimensional structured illumination microscopy (3D-SIM). Scale bars, 5 μm (*top panels*) and 500 nm (*middle and bottom panels* of 3D images). **h** The intensity plots of the rings in 3D images (**g**) from top and side views. **i** Representative images of RCTE cells stained with MIF antibody and co-stained with pericentrin (red). Scale bar, 5 μm. **j** Representative images of RCTE cells stained with MIF antibody and co-stained with acetylated α-tubulin (Ac-αTub) (red), which were visualized under 2D microscope and 3D-SIM microscopy. The “top or side” views are regarding the centriole structures. Scale bars, 5 μm (*top panels*) and 500 nm (*middle and bottom panels* of 3D images).

Cilia assembly and disassembly is a dynamic process during cell cycle, in that cells tend to assemble cilia during cellular quiescence (G0/early G1 phase) and disassemble cilia during S/G2/M phase, but almost invariably resorbed before mitotic entry, and to re-appear post-cytokinesis [17]. To investigate whether the expression of intracellular MIF changes during cell cycle, we treated human renal cortical tubular epithelial (RCTE) cells with thymidine-nocodazole or double thymidine block which could synchronize cells at G2/M or G1/S phase (Fig. 1a), respectively. After removal of these inhibitors, we collected cells at the different time points (Fig. 1b). The synchronization of cells with indicated inhibitors was confirmed by FACS analysis (Fig. 1c, d). We found that the expression of MIF, Cyclin B, Cdc20 and CDH1 were fluctuated in thymidine-nocodazole synchronized RCTE cells during cell cycle, which was peaked in G2/M phases, and then sharply reduced in G1 phase (Fig. 1e). When RCTE cells were synchronized with double thymidine block at G1/S phase, the expression of MIF as well as Cyclin B, Cdc20 and CDH1 was also peaked in G2/M phase and reduced in G1 phase during cell cycle (Fig. 1f). In addition, we found that the expression of MIF, Cyclin B, Cdc20 and CDH1 was also peaked in G2/M phases and reduced in G1 phase during cell cycle in Hela cells synchronized with either thymidine-nocodazole or double thymidine block (Supplementary information, Fig. S1a–d). The reduction of MIF at G1 phase and the increase of MIF at G2/M phase are associated with cilia assembly and disassembly during cell cycle, suggesting that MIF may play a role in these processes.

### MIF forms a ring-like structure at proximal end of the centrioles

**Fig. 2:**
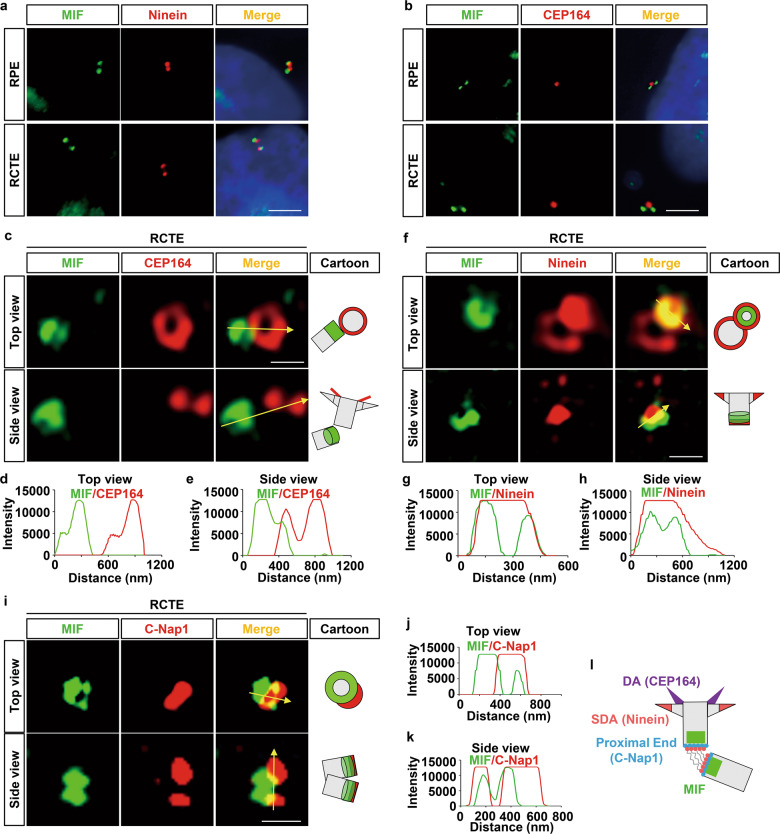
MIF is located and forms ring-like structures at the proximal ends of the centrioles. Representative images of RCTE cells stained with MIF antibody and co-stained with Ninein (**a**) or CEP164 (**b**) under 2D microscope. Scale bars, 5 μm. **c** Representative images of RCTE cells stained with MIF antibody and co-stained with CEP164 (red), which were visualized under 3D-SIM microscopy. Scale bars, 500 nm. The intensity plots of the rings in 3D images (**c**) from top (**d**)and side (**e**) views. **f** Representative images of RCTE cells stained with MIF antibody and co-stained with Ninein (red), which were visualized under 3D-SIM microscopy. Scale bars, 500 nm. The intensity plots of the rings in 3D images (**f**) from top (**g**) and side (**h**) views. **i** Representative images of RCTE cells stained with MIF antibody and co-stained with C-Nap1 (red), which were visualized under 3D-SIM microscopy. Scale bars, 500 nm. The intensity plots of the rings in 3D images (**i**) from top (**j**) and side (**k**) views. **l** Schematic of the localization of MIF, CEP164, Ninein, and C-Nap1 at centrosomes. The “top or side” views are regarding the centriole structures. DA distal appendage, SDA subdistal appendage.

To investigate whether MIF plays a role in cilia biogenesis, first, we determined the subcellular localization of MIF in RCTE and human retinal pigment epithelial (RPE) cells [18]. We found that MIF was localized at basal bodies (centrioles) marked with γ-tubulin and pericentrin but not along axoneme marked with α-acetyl-tubulin (Ac-αTub) in RCTE (Fig. 1g–j, Supplementary information, Fig. S2a) and RPE cells (Supplementary information, Fig. S1e–h, Fig. S2b, c). With quantification analysis, we found that MIF was located at centrioles in about 90% of RCTE and 89% of RPE cells (Supplementary information, Fig. S2). The localization of MIF at basal bodies was verified with a second MIF antibody in RCTE and RPE cells co-stained with γ-tubulin and Arl13b, another axoneme marker (Supplementary information, Fig. S1i, j). With 3D structured illumination microscopy (3D-SIM), we found that MIF formed a ring-like structure around the centrioles (both mother and daughter centrioles) in RCTE cells (Fig. 1g, j) and RPE cells (Supplementary information, Fig. S1e, h). The dots surrounding staining under 3D microscopy should be cytosolic MIF. We also found MIF was localized at basal bodies (centrioles) marked with γ-tubulin but not on axoneme marked with Ac-αTub in mouse embryonic fibroblasts (MEFs) after serum deprivation for 24 h (Supplementary information, Fig. S3). The basal body/centriole localization of MIF was further confirmed in vivo in renal tubular cells, marked with both DBA (collecting ducts marker) and LTL (proximal tubule marker) in *Mif* wild type kidneys (Supplementary information, Fig. S4). The loss of MIF staining at basal bodies/centrioles in renal tubular cells in *Mif* knockout kidneys supported the specificity of MIF antibody (Supplementary information, Fig. S4). To determine a more precise localization of MIF at centrioles, we co-stained MIF with different centriolar/centrosomal markers, including CEP164 (a marker for a distal appendage [19]), Ninein (a marker for sub-distal appendages of mother centrioles and proximal ends of both centrioles [20]) and γ-tubulin (a marker for pericentriolar materials) in RCTE cells. Under 2D-microscopy, MIF co-localized with all these markers, in that MIF was co-localized with γ-tubulin (Fig. 1g) and Ninein (Fig. 2a), and was localized adjacent to CEP164 (Fig. 2b). Under 3D-SIM, we found that MIF was not colocalized with CEP164 (Fig. 2c–e) in RCTE cells, suggesting that MIF was not localized at or around the distal appendages, whereas MIF was colocalized with Ninein around proximal end of mother and daughter centrioles (Fig. 2f–h) in those cells. To further support the localization of MIF at the proximal end of centrioles, we co-stained MIF with C-Nap1 (centrosomal NEK2-associated protein 1) that anchors rootletin and CEP68 filaments to the proximal ends of the two centrioles and is involved in the regulation of procentriole formation during late G1/S phase [21]. We found a ring-like structure of MIF surrounding the proximal end of centriole marked with C-Nap1 (Fig. 2i–k). These results suggested that MIF is a proximal end protein of both centrioles as indicated in the diagram (Fig. 2l).

### MIF is a critical regulator of cilia formation and elongation

**Fig. 3:**
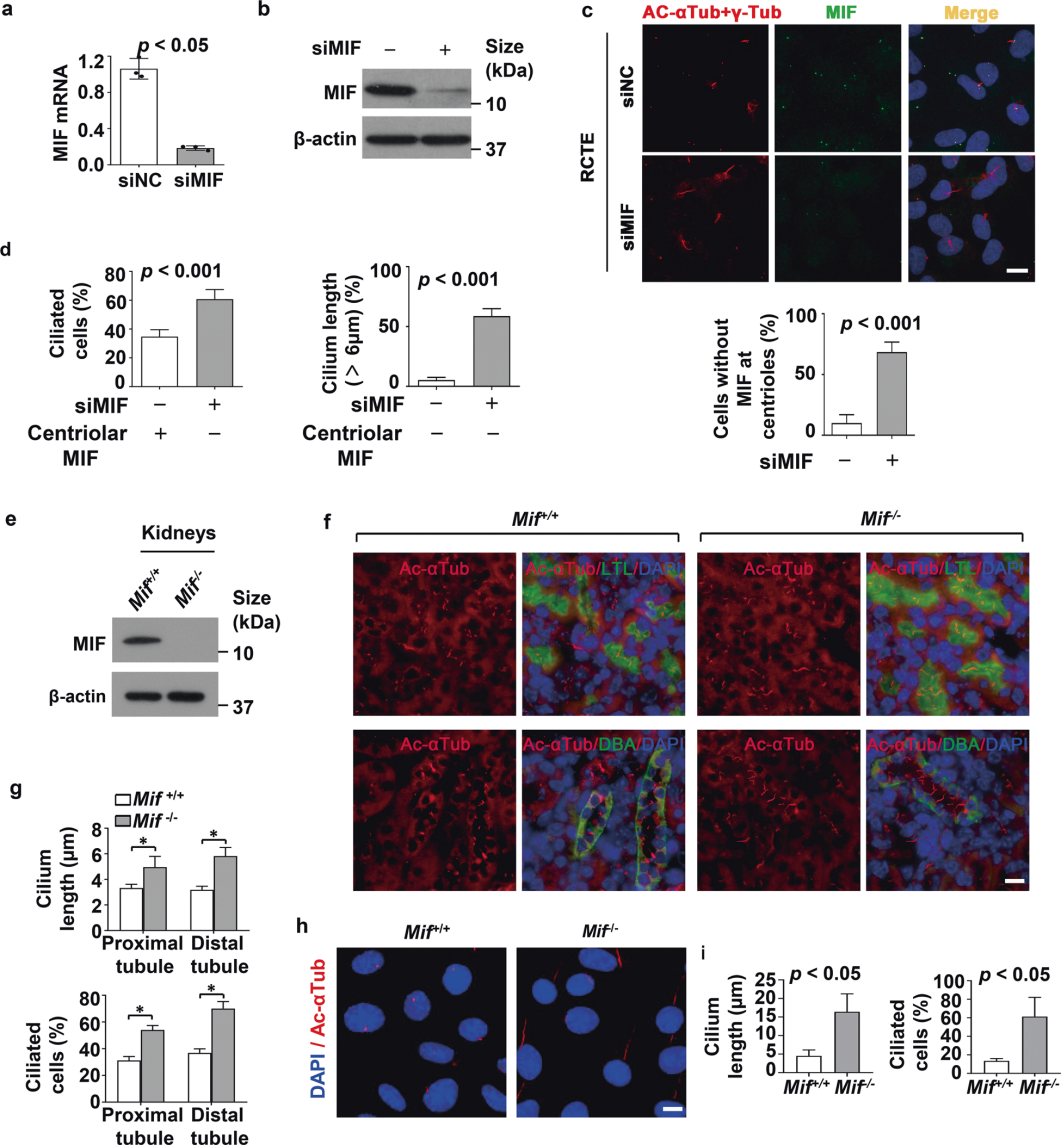
Depletion of MIF results in aberrant ciliogenesis. The levels of MIF in RCTE cells transfection with MIF and control siRNA were examined with qRT-PCR (**a**) and Western blot (**b**) analysis. **c** Representative images of RCTE cells transfected with MIF and control siRNAs for 48 h and serum starved for 24 h, and then stained with MIF (green) and Ac- αTub (red) and γ-Tub (red) antibodies. We quantified the percentage of cells without MIF at the centrioles in over 100 cells in indicated group, about 68.5% of cells were missing MIF at centrioles in MIF siRNA treated cells, whereas about 10% of control siRNA-treated cells had MIF at centrioles. Scale bars, 10 μm. **d** Knockdown of MIF in RCTE cells (**c**) increased the percentage of ciliated cells and cilia length. We quantified the percentage of ciliated cells and cilia length (>6 μm) in cells with missing MIF at centrioles. For each group, over 100 cells were counted. **e** Western blot analysis of Mif in kidneys from wild-type and *Mif* knockout mice (*n* = 5). **f** Representative images of kidney sections from 21 days old *Mif* ^*+/+*^ and *Mif* ^*–/–*^ mice (*n* = 5 mice for each group) stained with Ac-αTub (red) and DBA (dolichos biflorus agglutinin), a collecting duct marker and LTL (lotus tetragonolobus lectin), a proximal tubule marker, and as well as DAPI (blue). White dashed lines represent the border of each renal tubule. Scale bars, 10 μm. **g** Knockout of Mif in kidneys (**f**) increased cilia length (top) and the percentage of ciliated cells (bottom) in the lumens of renal tubules compared to the controls. **h** Representative images of primary renal tubule cells isolated from 21 days old *Mif* ^+/+^ and *Mif* ^–/–^ kidneys (n = 5 mice for each group) and stained with Ac- αTub (red) and DAPI (blue), about 50 proximal tubule or distal tubule/collecting duct cells were measured. Scale bars, 5 μm. **i** Knockout of Mif in primary renal tubular cells (**h**) increased cilia length (left) and the percentage of ciliated cells (right) compared to the controls, for (**g**) and (**i**), data are represented as the mean value ± s.d. *p* < 0.05 as determined by unpaired two-tailed Student’s *t* test.

To support a role of MIF on ciliogenesis, we found that knockdown of MIF (Fig. 3a, b; Supplementary information, Fig. S5a, b) increased the number of ciliated cells and cilia length in RCTE (Fig. 3c, d) and RPE (Supplementary information, Fig. S5c, d) cells compared to those in the control siRNA transfected cells, in which the efficiency of MIF knockdown with siRNA was about 78.28% as examined with qRT-PCR analysis. With this MIF knockdown efficiency, MIF lost its centriole localization in about 68.5% cells out of total cells treated with MIF siRNA, whereas only about 10% of control siRNA-treated cells could not detect MIF at centrioles (Fig. 3c). If we only counted the ciliated cells in those 68.5% cell population with missing MIF at centrioles, the percentage of ciliated cells was 60.67%, and if we only counted the ciliated cells with MIF at centrioles out of total viewed control siRNA treated cells, the percentage of ciliated cells was ~35% (Fig. 3d, *left panel*). Within the 68.5% of cells that loss MIF at centrioles, we found that about 59.5% of those cells showed an increase of cilia length (>6 μm), whereas only about 6% of control siRNA-treated cells showed a cilia length over 6 μm (Fig. 3d, *right panel*), which might be from those 10% cells without MIF at centrioles. Also, we found that treatment with MIF inhibitor, ISO-1, also increased the numbers of ciliated cells and cilia length in RCTE (Supplementary information, Fig. S5e, f) and RPE (Supplementary information, Fig. S5g, h) cells compared to those in control cells treated with DMSO, and 3) treatment with purified human recombinant MIF (rhMIF) decreased the numbers of ciliated cells and cilia length in RCTE cells compared to that in control cells treated with PBS (Supplementary information, Fig. S5i, j).

To elucidate the role of MIF in ciliogenesis in vivo, we examined ciliogenesis in cilia-rich mouse kidneys. With the co-staining of MIF with DBA or LTL, we found that knockout of *Mif* (Fig. 3e) strikingly increased cilia length and ciliated cells in renal tubular cells in kidneys collected from 21 days (Fig. 3f, g) and 3 months old *Mif* knockout mice (Supplementary information, Fig. S6a, b) compared to those in the renal tubular cells in kidneys from age matched *Mif* wild type mice. We further found that knockout of *Mif* increased cilia length and ciliated cells in primary renal epithelial cells isolated from *Mif* knockout kidneys compared to that in *Mif* wild-type renal epithelial cells isolated from age-matched wild type kidneys (Fig. 3h, I; Supplementary information, Fig. S6c, d). These results suggest that MIF functions as a critical regulator of cilia biogenesis in vitro and in vivo.

### MIF blocks cilia elongation via inhibiting CP110 removal, TTBK2 recruitment and the accumulation of CEP290 at centriolar satellites

**Fig. 4:**
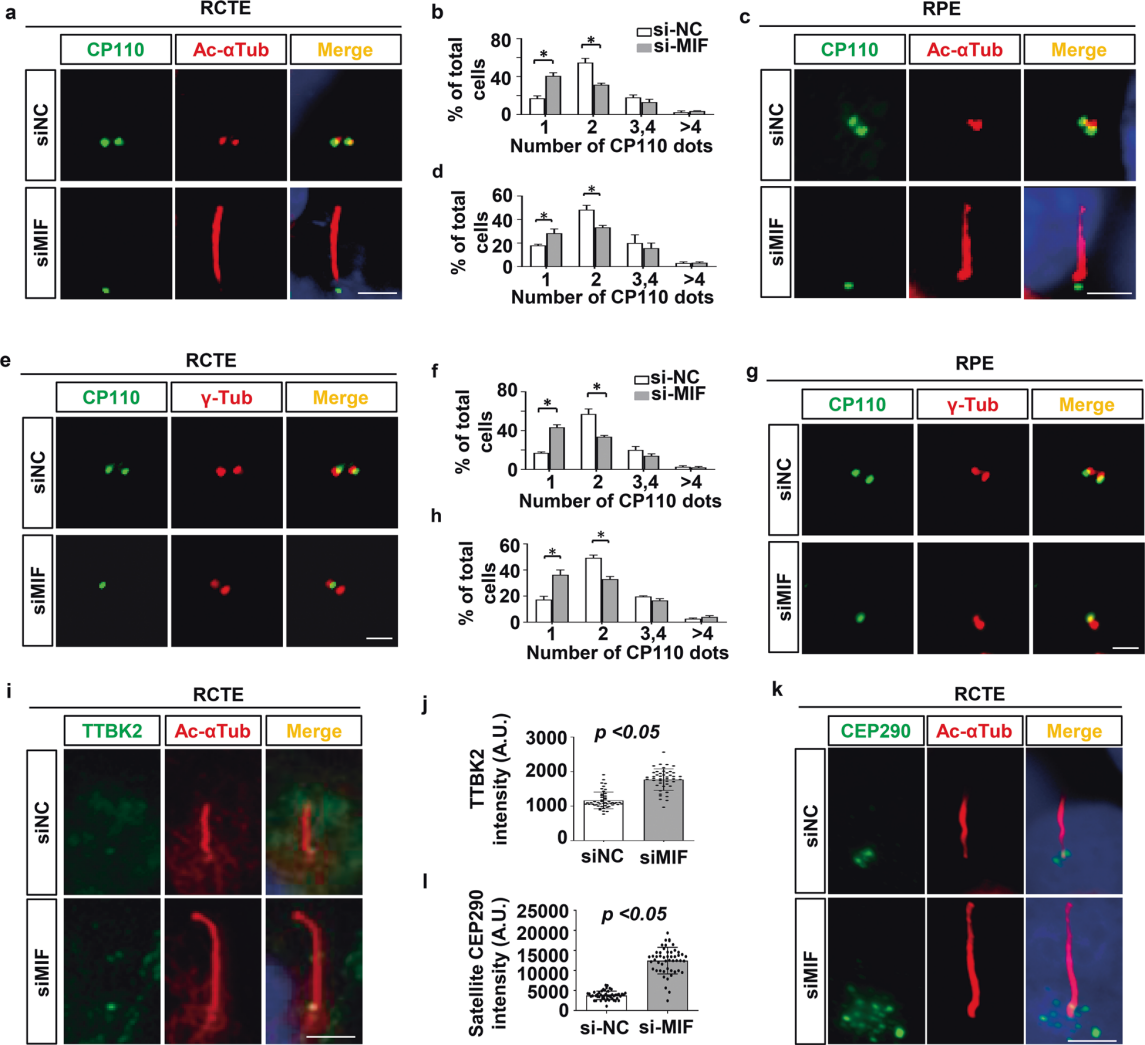
Depletion of MIF increases the removal of CP110 from mother centriole, the recruitment of TTBK2 to basal body and the accumulation of CEP290 at centriolar satellites. Representative images of RCTE (**a**) and RPE (**c**) cells transfected with MIF or control siRNAs for 48 h without starvation, and then stained with CP110 (green) and Ac- αTub (red) antibodies and co-stained with DAPI (blue). Scale bars, 5 μm. **b**, **d**Quantification of the percentage of cells with the indicated numbers of CP110 dots at centrioles in MIF knockdown RCTE (**a**) and RPE (**c**) cells from three independent experiments. *p < 0.05. Representative images of RCTE (**e**) and RPE (**g**) cells transfected with MIF and control siRNAs for 48 h without starvation, and then stained with CP110 and γ-tubulin (red) antibodies. Scale bars, 5 μm. **f**, **h** Quanti**f**ication of the percentage of cells with the indicated numbers of CP110 dots at centrioles in MIF knockdown RCTE (**e**) and RPE (**g**) cells from three independent experiments. *p < 0.05. **i** Representative images of RCTE cells transfected with MIF and control siRNAs for 48 h and serum starved for 24 h, then stained with TTBK2 and Ac- αTub (red) antibodies and co-stained with DAPI (blue). Scale bars, 5 μm. **j** Quantifications of TTBK2 signals at basal bodies in (**i**) (*n* = 50 cells for each group). **k** Representative images of RCTE cells transfected with MIF and control siRNAs for 48 h and serum starved for 24 h, then stained with CEP290 and Ac-αTub (red) antibodies and co-stained with DAPI (blue). Scale bars, 5 μm. **l** Quantifications of CEP290 signals at centriolar satellites in (**k**) (*n* = 50 cells for each group). Statistics were performed using student’s *t* test, *p < 0.05.

Cilia assembly and elongation require the removal of the CP110, a centrosomal protein, from the distal end of mother centriole to allow the growth of axonemal microtubules [22]. We found that depletion of MIF resulted in the removal of CP110 from mother centriole in RCTE cells (Fig. 4a, b) and RPE cells (Fig. 4c, d) compared to its presence on both centrioles in the control cells. We further found that depletion of MIF did not affect the localization of γ-tubulin, which was detected on both centrioles as marked as two dots in RCTE cells (Fig. 4e, f) and RPE cells (Fig. 4g, h), suggesting that MIF depletion did not disturb centrosomes in those cells. The kinase TTBK2 mediates the removal and dissociation of CP110 from the CP110 and CEP290 complex at the mother centriole, leading to the formation of the primary cilium. We found that knockdown of MIF increased the recruitment of TTBK2 to basal body in RCTE cells (Fig. 4i, j). It has been reported that the interaction of CP110 with CEP290 antagonizes the action of CEP290, which in turn prevents ciliogenesis [23]. We found that knockdown of MIF resulted in an increase of centriolar satellite-associated CEP290 around basal bodies in RCTE cells (Fig. 4k, l). These results suggest that MIF may control cilia assembly and elongation by increasing the recruitment of CP110 to the mother centriole and decreasing the recruitment of TTBK2 to basal bodies and the accumulation of CEP290 at centriolar satellites.

### Loss of MIF promotes IFT related proteins recruitment and entry

**Fig. 5:**
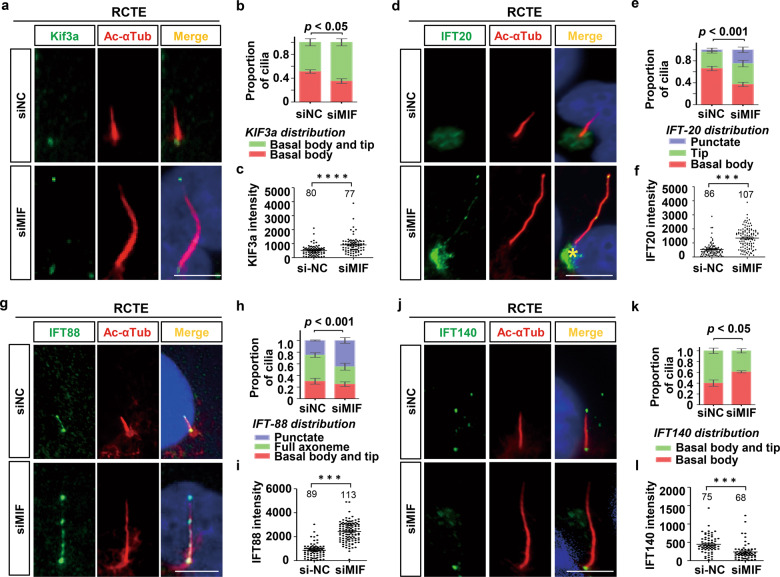
Knockdown of MIF affects the trafficking of KIF3a and IFT particles, including IFT20, IFT88 and IFT140. **a** Representative image of RCTE cells transfected with MIF and control siRNAs for 48 h and serum starved for 24 h, then stained with KIF3a and Ac-αTub (red) antibodies and co-stained with DAPI (blue). Scale bars, 5 μm. **b** Stacked bar graph represented KIF3a distribution at primary cilia in RCTE cells transfected with MIF and control siRNAs. **c** Quantification of the intensity of KIF3a signals in RCTE cells transfected with MIF and control siRNAs. **d** Representative images of RCTE cells transfected with MIF and control siRNAs for 48 h and then serum starved for 24 h, and stained with IFT20 and Ac-αTub(red) antibodies and co-stained with DAPI (blue). Scale bars, 5 μm. **e** Stacked bar graph represented IFT20 distribution at primary cilia in RCTE cells transfected with MIF and control siRNAs. **f** Quantification of the intensity of IFT20 signals in RCTE cells transfected with MIF and control siRNAs. **g** Representative images of RCTE cells transfected with MIF and control siRNAs for 48 h and then serum starved for 24 h, and stained with IFT88 and Ac-αTub(red) antibodies and co-stained with DAPI (blue). Scale bars, 5 μm. **h** Stacked bar graph represented IFT88 distribution at primary cilia in RCTE cells transfected with MIF and control siRNAs. **i** Quantification of the intensity of IFT88 signals in RCTE cells transfected with MIF and control siRNAs. **j** Representative images of RCTE cells transfected with MIF and control siRNAs for 48 h and then serum starved for 24 h, and stained with IFT140 and Ac-αTub(red) antibodies and co-stained with DAPI (blue). Scale bars, 5 μm. **k** Stacked bar graph represented IFT140 distribution at primary cilia in RCTE cells transfected with MIF and control siRNAs. **l** Quantification of the intensity of IFT140 signals in RCTE cells transfected with MIF and control siRNAs. Numbers cells analyzed are indicated in the bars or above each dataset, for each group. Statistics were performed using one-sided one-way ANOVA followed by post hoc LSD test (**b,** **e,** **h,** **k**). Statistics were performed using student’s *t*-test (**c,** **f,** **i,** **l**). ***p < 0.001.

We further found that knockdown of MIF and inhibition of MIF with ISO-1 resulted in a cilia tip accumulation of KIF3a, an essential subunit of the kinesin II motor that is required for cilia formation in nearly all cells [24], in RCTE cells, which was in contrast to its basal body localization in control cells (Fig. 5a–c; Supplementary information Fig. S7a–c). The accumulation of KIF3a at ciliary tips suggested an increase of anterograde trafficking of cilia proteins, which is regulated by IFT B complex, and a decrease of retrograde trafficking of these proteins, which is regulated by IFT A complex, in MIF knockdown cells. To support this, we found that knockdown of MIF and inhibition of MIF with ISO-1 resulted in an increase of the accumulation of IFT20 at the cilia tips in RCTE cells (Fig. 5d–f; Supplementary information Fig. S7d–f). We also found that knockdown of MIF and inhibition of MIF with ISO-1 increased the intensity of IFT88 at ciliary tips, resulting in a punctate distribution of IFT88 along cilia in RCTE cells (Fig. 5g–I; Supplementary information Fig. S7g–i). Both IFT20 and IFT88 belong to IFT B complex. In addition, knockdown of MIF and inhibition of MIF with ISO-1 decreased the tip location of a core IFT A protein, IFT140, in RCTE cells (Fig. 5j–l; Supplementary information Fig. S7j–l). Unexpectedly but importantly, we found that knockdown of MIF (Supplementary information, Fig. S8a–c) and inhibition of MIF with ISO-1 (Supplementary information, Fig. S8d–f) increased the mRNA and protein levels of KIF3a and IFT20 in RCTE cells. These results suggest that MIF regulates the trafficking of KIF3a and IFT proteins, and may also negatively regulate the transcription of KIF3a and IFT20 and other genes related to ciliary biogenesis.

### Identification of MIF target genes with ChIP-sequencing (ChIP-seq) analysis

**Fig. 6:**
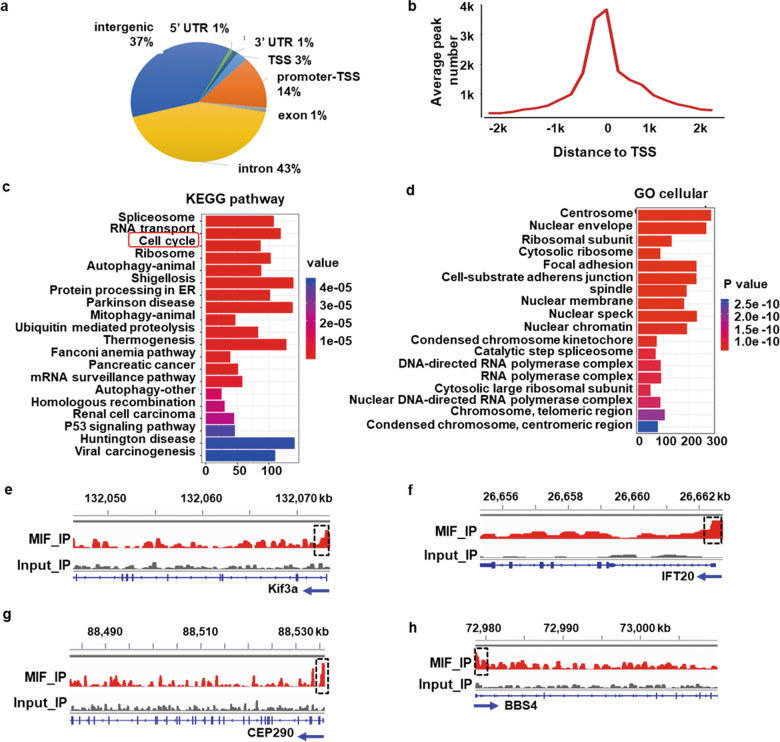
Identification of MIF target genes with ChIP-seq analysis. **a** The distribution of MIF ChIP-peaks was across different genomic regions. The pie chart shows that MIF binds to the promoters-TSS (transcription start site) region in about 14% of its target genes. **b** The distribution of promoter peaks identified with MIF ChIP-seq was markedly concentrated near the TSS. **c,** **d** KEGG pathway and GO categories analysis of the MIF target genes. Peaks of MIF occupancy on the promoters of *KIF3a* (**e**)*, IFT20* (**f**)*, CEP290* (**g**) *and BBS4* (**h**) were identified by manual inspection and were shown as the input and MIF ChIP.

To support that MIF could regulate the expression of ciliary genes, we found that knockdown of MIF increased the transcription of genes of IFT A particles (IFT121, IFT139, IFT140, IFT144), IFT B particles (IFT27, IFT88), centriolar satellite/transition zone protein (CEP290), transition zone protein (TTBK2), distal appendage protein (CEP89), basal body and axoneme protein (Rab8a), basal body protein (OFD1) and BBSome protein (BBS4) (Supplementary information Fig. S9). To understand how MIF regulates the transcription of ciliary genes, we searched of public available database (GSE65110) [13], and identified the genome-wide MIF target genes with ChIP-seq analysis. We found that there were about 14% of reproducible and statistically significant MIF binding peaks located within gene promoter regions (0–1000 bp) (Fig. 6a), which were markedly concentrated near the transcription start sites (TSS) (Fig. 6b). With Kyoto Encyclopedia of Genes and Genomes (KEGG) pathway-based analysis, we found that the identified MIF target genes are associated with diverse signaling pathways, including spliceosome, RNA transport, cell cycle, ribosome, autophagy, protein processing in endoplasmic reticulum, Parkinson disease, mitophagy, ubiquitin mediated proteolysis, etc. (Fig. 6c). With GREAT (Genomic Regions Enrichment of Annotations Tool) analysis, a tool that allows us to find enriched ontological terms in a set of genomic regions to better understand the underlying biological processes, we clustered MIF occupied genes with a range of gene ontology (GO) biological processes, including autophagy, cell cycle transition signaling, and cell cycle checkpoint pathways, etc. (Supplementary information, Fig. S10). The GO cellular component analysis also revealed that MIF target genes were highly enriched for centrosome, and other cellular structures, such as nuclear envelope, nuclear membrane, nuclear speck and nuclear chromatin, etc. (Fig. 6d). We summarized the potential MIF target genes related to cell cycle and ciliogenesis as well as other cellular activity in Supplementary Data 1 and 2. As an example, we showed the identified peaks in ChIP-seq analysis corresponding to the binding of MIF on the promoters of KIF3a, IFT20, CEP290 and BBS4 genes (Fig. 6e–h, *black boxes*), which was confirmed with ChIP assay (Supplementary information, Fig. S11a–d). These results suggest that MIF is able to function as a novel transcriptional factor to regulate the expression of genes associated with cell cycle and cilia biogenesis through directly binding on the promoters of those genes.

### The translocation of MIF to nucleus is dependent on its interaction with 14-3-3

**Fig. 7:**
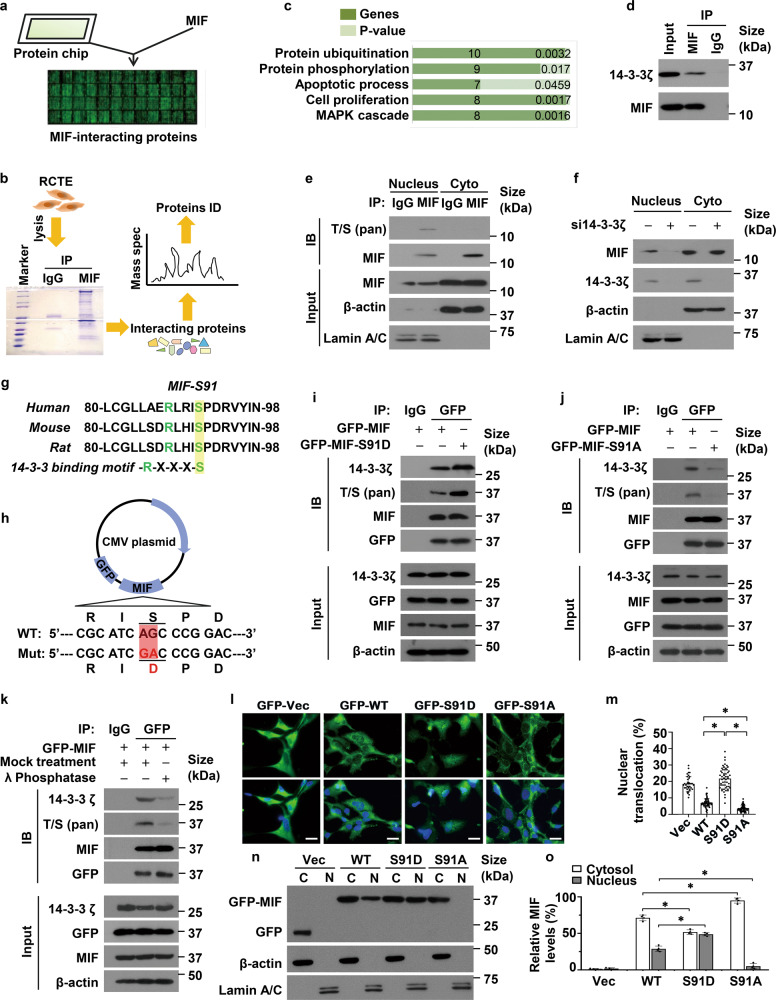
The translocation of MIF to nucleus is dependent on its phosphorylation and interaction with 14-3-3ζ. **a** Identification of MIF-associated proteins by protein chip. **b** Identification of MIF-associated proteins by immunoprecipitation with MIF antibody and followed by mass spectrometry (MS) in RCTE cells. **c** MIF associated proteins identified by MS were clustered with a range of gene ontology (GO) biological processes by functional annotation of MIF binding sites using the Genomic Regions Enrichment of Annotations Tool (GREAT). **d** Immunoprecipitation assay was performed to detect the interaction between MIF and 14-3-3ζ in RCTE cells. **e** The nuclear and cytoplasmic fractions were isolated from RCTE cells and then those fractions were subjected to immunoprecipitation with MIF antibody and IgG, and followed by immunoblotting with indicated antibodies. **f** Western blot analysis of MIF and 14-3-3ζ in nuclear and cytoplasmic fractions isolated RCTE cells transfected with 14-3-3ζ and control siRNAs. **g** Alignment of MIF sequences from different species (Human, Mouse, Rat). Highlighted letters indicate the conserved canonical 14-3-3ζ binding motif. **h** The schematic diagram shows the mutation of S91D in GFP tagged MIF plasmid**. i** Immunoprecipitation assay to detect the phosphorylation of MIF and the interaction between GFP tagged MIF and 14-3-3ζ in RCTE cells transfected with GFP tagged wild type MIF and GFP tagged mutant MIF-S91D plasmids. **j** Immunoprecipitation assay to detect the phosphorylation of MIF and the interaction between GFP tagged MIF and 14-3-3ζ in RCTE cells transfected with GFP tagged wild type MIF and GFP-tagged mutant MIF-S91A plasmids for 24 h. **k** Immunoprecipitation assay to detect the phosphorylation of MIF and the interaction between GFP tagged MIF and 14-3-3ζ in RCTE cells treated with λ-phosphatase and vehicle (control). **l** Representative images of HEK293 cells transfected with GFP-tagged wild-type MIF and GFP-tagged mutant MIF (S91D or S91A) or GFP-Vector. Scale bars, 10 μm. **m** Quantification of the percentage of cells with nuclear translocation of MIF in HEK293T cells transfected with GFP-tagged wild type MIF plasmid and GFP-tagged mutant MIF (S91D or S91A) plasmid or GFP-vector. **n** Western blot analysis of GFP-tagged MIF in nuclear (N) and cytoplasmic (C) fractions isolated HEK293T cells transfected with GFP-tagged wild type MIF (WT) plasmid and GFP-tagged mutant MIF (S91D or S91A) plasmid. **o** Statistical analysis of relative levels of MIF in cytosol and nuclear fractions isolated HEK293T cells transfected with GFP-tagged wild-type MIF and GFP-tagged mutant MIF (S91D or S91A) from three independent experiments. All data are represented as the mean value ± s.d. Significant differences were identified by student’s *t* test. *p < 0.05.

In order to act as a transcriptional factor, MIF has to be translocated from cytosol to nucleus. To identify a MIF binding partner that might facilitate its translocation into nucleus, we performed protein chip by using a human recombinant MIF protein to screen its interacting proteins on HuProtTM human protein chip which contains more than 21,000 human recombinant proteins (*See Methods*) (Fig. 7a). In brief, recombinant human MIF (rhMIF) or blocking buffer was probed on the human protein chip, and then the microarray was further incubated with a Cy3-conjugated antibody to present the MIF-protein interactions. With this analysis, we identified 225 proteins as potential binding partners of MIF (Supplementary information, Data 3).

In addition, we identified the proteins that interacted with MIF by performing immunoprecipitation with MIF antibody and followed by mass spectrometry (MS) analysis in human renal cortical tubular epithelial (RCTE) cells (Fig. 7b). With this analysis we identified 512 proteins as potential binding partners of MIF (Supplementary Data 4), including YWHAZ (14-3-3ζ), phosphatidylinositol-5-phosphate 4-kinase type 2 alpha (PIP4K2a), etc. Functional annotation analysis revealed that the MIF interaction proteins were significantly enriched for proteins involved in protein ubiquitination (*p* < 0.0032), protein phosphorylation (*p* < 0.017), apoptotic process (*p* < 0.0459), and MAPK cascade (*p* < 0.0016), many of which affect protein activity (Fig. 7c).

14-3-3 is highly conserved and can be self-assembled into homo- and heterodimers with a diverse array of cellular proteins, including transcription factors, biosynthetic enzymes, cytoskeletal proteins, apoptosis factors and tumor suppressors [25]. We confirmed that MIF interacted with 14-3-3ζ in RCTE cells by co-IP assay (Fig. 7d). We found that MIF could be detected in nuclear and cytosol fractions in RCTE cells (Fig. 7e), whereas knockdown of 14-3-3ζ with siRNA decreased the levels of MIF in nuclear fraction but not that in cytosol fraction (Fig. 7f), suggesting that the binding of MIF with 14-3-3ζ might promote it nuclear transportation.

With the alignment of MIF amino acid sequence with a conserved 14-3-3 binding motif, RXXXS [26], we identified that RXXXS91 of MIF is a potential 14-3-3 binding site (Fig. 7g). It has been reported that the phosphorylation of serine in RXXXS motif facilitates the nuclear translocation of 14-3-3 partner(s). To test this possibility, we generated MIF mutant constructs by replacing the serine 91 of MIF with aspartic acid (D) (constantly phosphorylated mutation) or alanine (A) (un-phosphorylated mutation) (Fig. 7h). Due to that a phospho-MIF antibody is not available, to detect the phosphorylation of MIF, we used a MIF antibody to pull down MIF and then detected the phosphorylation of MIF with an anti-pan-phospho-threonine/serine (phospho-T/S) antibody. We found that the interaction between GFP tagged mutant MIF (S91D) and 14-3-3ζ was increased compared to that between GFP tagged wild type MIF and 14-3-3ζ in RCTE cells (Fig. 7i). However, we found a negligible interaction between GFP tagged mutant MIF (S91A) and 14-3-3ζ compared to the interaction between GFP tagged wild type MIF and 14-3-3ζ (Fig. 7j). Treatment with lambda phosphatase also decreased the interaction of GFP-tagged wild type MIF with 14-3-3ζ (Fig. 7k). These results suggested that the interaction between MIF and 14-3-3ζ might be dependent on the phosphorylation of MIF at S91.

### Phosphorylation of MIF at Ser91 affects its nuclear translocation

To investigate whether phosphorylation of MIF at S91 is involved in its nuclear translocation/exportation, we transfected GFP-tagged wild type MIF and mutant MIF (S91D or S91A) into HEK293T cells. We found that GFP tagged wild-type MIF and mutant MIF at S91A was mostly located in cytosol, whereas the GFP tagged MIF S91D mutant was located in both nucleus and cytosol in GFP positive cells as examined with immunostaining analysis (Fig. 7l, m). We further found that the level of the GFP tagged mutant MIF at S91D was increased in nuclear fraction compared to that in nuclear fraction of cells transfected with GFP tagged wild-type MIF (Fig. 7n, o), whereas the nuclear transportation of mutant MIF S91A was blocked (Fig. 7l, n). These results support that the phosphorylation of MIF at Ser91 is required for MIF nuclear translocation.

In addition, we found that the expression of cilia associated genes, including KIF3A, IFT20, CEP290 and BBS4, and the percentage of ciliated cells and cilia length were significantly decreased in RCTE (Supplementary information, Fig. S12a–g) and RPE (Supplementary information, Fig. S13a–g) cells transfected with GFP tagged wild type MIF and MIFS91D compared to those in cells transfected with GFP-vector and GFP tagged MIFS91A. These results support that the phosphorylation of MIF at Ser91 is required for MIF nuclear translocation and MIF mediated ciliary gene expression and cilia biogenesis.

### PIP4K2a interacts with MIF and induces the phosphorylation of MIF at Ser91

**Fig. 8:**
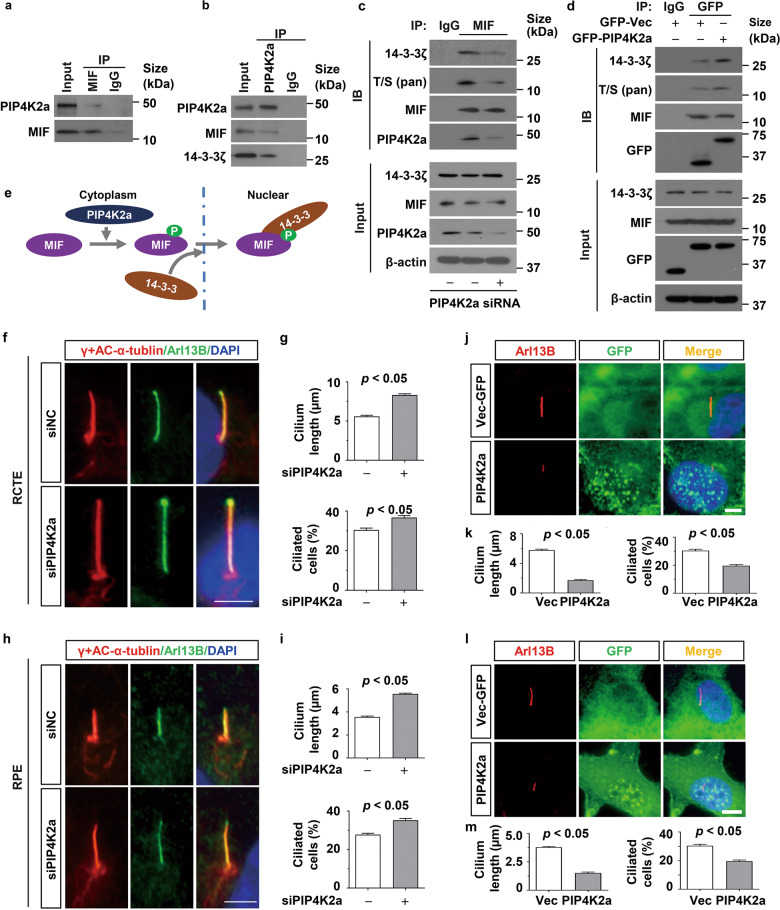
Phosphorylation of MIF at Ser91 is regulated byPIP4K2a. Lysates from RCTE cells were subjected to immunoprecipitation (IP) with anti-MIF (**a**) and anti-PIP4K2a (**b**) antibodies, and immunoblotting with the indicated antibodies. **c** Immunoprecipitation assay to detect the phosphorylation of MIF and the interaction between MIF and 14-3-3ζ in RCTE cells transfected PIP4K2a and control siRNAs. **d** Immunoprecipitation assay to detect the phosphorylation of MIF and the interaction between GFP-MIF and 14-3-3ζ in RCTE cells transfected with GFP-Vector plasmids and GFP tagged PIP4K2a plasmids. **e** Schematic diagram indicates that PIP4K2a phosphorylates MIF at S91 and phospho-MIF interacts with 14-3-3ζ to promote the nuclear translocation of MIF. **f** Representative images of RCTE cells transfected with PIP4K2a and control siRNAs for 48 h and then serum starved for 24 h, and stained with ARL13B, γ-tubulin and Ac-αTub(red) antibodies and co-stained with DAPI (blue). Scale bars, 5 μm. **g** Knockdown of PIP4K2a in RCTE cells (**f**) increased cilia length (top) and the percentage of ciliated cells (bottom). **h** Representative images of RPE cells transfected with PIP4K2a and control siRNAs for 48 h and serum starved for 24 h, and then stained with ARL13B, γ-tubulin and Ac-αTub(red) antibodies and co-stained with DAPI (blue). Scale bars, 5 μm. **i** Knockdown of PIP4K2a in RPE cells (**h**) increased cilia length (top) and the percentage of ciliated cells (bottom). **j** Representative images of RCTE cells transfected with GFP-vector and GFP tagged PIP4K2a plasmids for 48 h and then serum starved for 24 h, and stained with ARL13B (red) and GFP antibodies and co-stained with DAPI (blue). Scale bars, 5 μm. **k** Overexpression of GFP tagged PIP4K2a in RCTE cells decreased cilium length (left) and percentage of ciliated cells compared to those in cells transfected with GFP-vector. **l** Representative images of RPE cells transfected with GFP-vector and GFP tagged PIP4K2a plasmids for 48 h and then serum starved for 24 h, and stained with ARL13B (red) and GFP antibodies and co-stained with DAPI (blue). Scale bars, 5 μm. **m** Overexpression of GFP tagged PIP4K2a in RPE cells decreased cilium length (left) and percentage of ciliated cells compared to those in cells transfected with GFP-vector. The percentages of ciliated cells in about 50 views were analyzed in each group, and the cilia lengths of about 100 cells were analyzed in each group (**g,** **k,** **i,** **m**). All statistical analysis was represented as the mean value ± s.d. Significant differences were identified by student’s *t* test.

Our protein chip and mass spectrometry (MS) analysis identified that PIP4K2a is a potential kinase responsible for the phosphorylation of MIF. To support this hypothesis, we confirmed that PIP4K2a interacted with MIF and 14-3-3ζ in RCTE cells as examined with co-IP assay (Fig. 8a, b). We further found that knockdown of PIP4K2a decreased the phosphorylation of MIF, and decreased the interaction of MIF with 14-3-3ζ in RCTE cells (Fig. 8c), whereas overexpression of GFP tagged PIP4K2a increased the phosphorylation of MIF and the interaction of MIF with 14-3-3ζ in RCTE cells (Fig. 8d), suggesting a crosstalk between PIP4K2a and MIF as well as phospho-MIF and 14-3-3ζ in MIF nuclear translocation (Fig. 8e).

Knockdown and overexpression of PIP4K2a has no effect on the expression of MIF in RCTE (Supplementary information, Fig. S14a, b) and RPE cells (Supplementary information, Fig. S15a, b). However, knockdown PIP4K2a increased the transcription of ciliary genes, including KIF3a, IFT20, IFT88, TTBK2, CEP290 and BBS4 in RCTE (Supplementary information, Fig. S14a) and RPE cells (Supplementary information, Fig. S15a), and overexpression of PIP4K2a decreased the transcription of those ciliary genes in RCTE (Supplementary information, Fig. S14b) and RPE cells (Supplementary information, Fig. S15b). Importantly, we found that knockdown of PIP4K2a with siRNA also increased cilia length and ciliated cell population in RCTE cells (Fig. 8f, g) and RPE cells (Fig. 8h, i), whereas overexpression of GFP tagged PIP4K2A decreased cilia length and ciliated cell population in RCTE cells (Fig. 8j, k) and RPE cells (Fig. 8l, m). These results suggest that PIP4K2a is an upstream regulator of MIF, which may increase its interaction with 14-3-3ζ, resulting in its nuclear translocation to execute it transcriptional function to regulate cilia biogenesis.

## Discussion

**Fig. 9:**
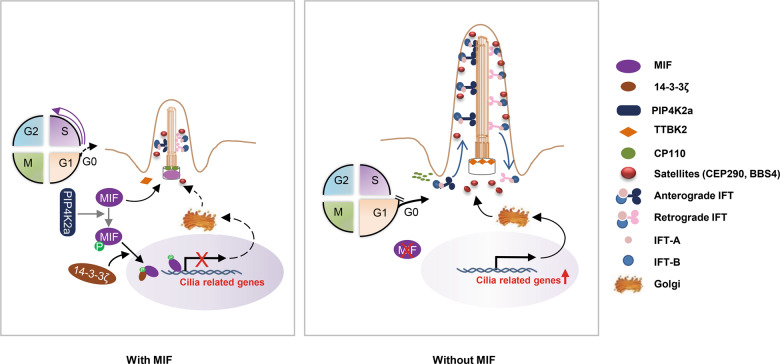
Working model. MIF is localized at basal body, which forms a ring like structure at proximal ends of centrioles in mammalian cells MIF regulates cilia assembly and elongation via affecting, 1) the removal of CP110 from mother centriole and the recruitment of TTBK2 to basal body, 2) the accumulation of CEP290 and BBS4 at centriolar satellites, 3) the trafficking of KIF3a and IFT particles, including IFT20, IFT88 and IFT140 (*left)*. Depletion of MIF promotes cilia initiation through TTBK2-CP110-CEP290-BBS4 axis and cilia elongation through an increase of the trafficking and entry of KIF3a, IFT20 and IFT88 into axoneme and a decrease of IFT140 at cilia tip (*right*). Importantly, MIF can be phosphorylated by PIP4K2a and phospho-MIF interacts with 14-3-3ζ, both of which facilitate the translocation of MIF into nucleus (*left*). The nuclear MIF binds to the promoters of its target genes, such as genes associated with cilia biogenesis, to negatively regulate their transcription. Thus, depletion of MIF may also promote cilia biogenesis through releasing its inhibition on the repression of ciliary genes. During the cell cycle, the expression of MIF was decrease in G1/G0 phase, which may contribute to cilia assembly, whereas in S phase MIF mediated repression of cilia genes should facilitate cilia disassembly and cell cycle progression.

MIF is expressed broadly in different types of human cells and has been related to different human disease, which has emerged as a promising drug target in diseases including sepsis, rheumatoid arthritis, polycystic kidney disease (PKD) and cancer. In addition to be an inflammatory cytokine, MIF also functions as an intracellular protein with different enzymatic activity. In this study, we identify unknown roles of intracellular MIF in cilia biogenesis and transcriptional regulation in homeostasis. We show that MIF is a novel centriole protein which forms a ring like structure at proximal end of centrioles to regulate cilia biogenesis through regulating the recruitment and removal of TTBK2 and CP110 to basal body, the accumulation of CEP290 at centriolar satellites, and the trafficking of IFT related proteins. We also show that MIF is a novel transcriptional factor that regulate the expression of genes related to cilia biogenesis and other signaling pathways. In addition, we identified that PIP4K2a is an upstream regulator of MIF, which phosphorylates MIF at S91 to promote the interaction of MIF with 14-3-3ζ and the translocation of MIF to nucleus where MIF functions as a transcriptional factor (Fig. 9).

The first key feature of this study is to identify MIF as a proximal end protein of centrioles to govern centriolar structure and control a balance between ciliary assembly and disassembly through defined ciliary proteins, including CP110, TTBK2, CEP290, IFT and IFT related proteins. CP110 has been recognized as a crucial and negative regulator of cilia biogenesis [22]. In response to cell cycle exit signals, CP110 is removed selectively from the mother centriole and degraded through ubiquitylation, which is an essential event for the quiescence-induced ciliogenesis [27]. The removal of CP110 coincides with the presence of TTBK2 at mother centriole [28]. It has been reported that TTBK2 promotes the removal of CP110 and promotes the recruitment of IFT proteins to build the ciliary axoneme, which is also one of the earliest identified events in ciliogenesis [28]. Our results that MIF is required for CP110 to the distal ends of the mother centrioles and depletion of MIF increases the recruitment of TTBK2 to basal body and perturbs centriolar localization of CP110 in mammalian cells suggests that MIF is an upstream regulator of TTBK2-CP110 signaling and plays an important role in TTBK2-CP110 mediated early steps of ciliogenesis.

Interestingly, treatment with MIF inhibitor, ISO-1, depleted MIF from centrioles. ISO-1 as a MIF antagonist can inhibit the D-dopachrome tautomerase activity of MIF through binding to the active site of residue Asn-97 of MIF: Protein data bank,1LJT is the name of ISO-1 crystal structure.), resulting in structural changes of MIF to block its binding with its partners, such as CD74 [29, 30]. The possible mechanisms that treatment with ISO-1 disrupts MIF centriole localization include that, 1) ISO-1 mediated the structural changes of MIF may disrupt its association with other proteins at centrioles, resulting in the loss of MIF from centrioles, and/or 2) treatment with ISO-1 decreases the levels of MIF (Fig. S8f), which has been reported in literatures [9, 31] and can be regulated by an auto-amplifying feedback loop between MIF/CD74 and HIF-1a/b [32] as well as by ICBP90 (also known as UHRF1) [33]. We propose that these two possibilities either work alone or together to disrupt the centriole localization of MIF when the cells are treated with ISO-1.

The second key feature of this study is to identify MIF as a transcriptional factor. MIF has been reported to regulate diverse intracellular signaling pathways, including ERK/MAPK, PI3K/Akt, NF-κB and p53-dependent pathway [9, 16, 34, 35], mainly via binding on its membrane receptors [35] to regulate cell proliferation and survival [36]. In addition, Src tyrosine kinase mediated the serine phosphorylation of CD74 is required for MIF-induced ERK1/2 kinase phosphorylation [37]. We have reported that upregulated MIF through CD74 to activate ERK, mammalian target of rapamycin (mTOR) and Rb-E2F signaling to regulated cystic renal epithelial cell proliferation and to suppress p53 signaling dependent cystic renal epithelial cell apoptosis in autosomal dominant polycystic kidney disease (ADPKD) [9]. Inhibition of MIF could also inactivate CD74-NF-κB signaling to protect kidneys against acute kidney injury [38]. We show here that MIF can transcriptionally target genes associated with diverse signaling pathways, suggesting a direct link of intracellular MIF with different intracellular signaling pathways in homeostasis and disease progression. In particular, the MIF target genes in cell cycle regulation may provide another potential mechanism to support our finding that MIF is involved in the regulation of cystic renal epithelial cell proliferation, leading to cyst growth in kidneys [9]. The role of MIF as a transcriptional factor in the regulation of genes related to ciliopathies [9] provides one of the mechanisms of how MIF regulates cilia biogenesis and also suggests a novel therapeutic potential by targeting MIF in the treatment of ciliopathy-associated diseases.

The third key feature of this study is to identify PIP4K2a as an upstream regulator of intracellular MIF. Although the activity of MIF can be regulated with its phosphorylation [39], however, the upstream kinase that regulate MIF phosphorylation is unknown. With the analyses of protein chip as well as immunoprecipitation and mass spectrometry, we identified several kinases, including PIP4K2a, PRKD2, TLK1 and BRAF, that may be responsible for MIF phosphorylation. We focus on PIP4K2a based on the report that PIP4K2a facilitates the conversion of phosphorylation of phosphatidylinositol 5-phosphate (PtdIns5P) to generate various phosphoinositide species, such as phosphatidylinositol 4,5 bis-phosphate (PI(4,5)P_2_) [40]. Cellular PtdIns5P could be produced by D-5-phosphorylation of phosphatidylinositol or by dephosphorylation of phosphatidylinositol 3,5 bis-phosphate (PI(3,5)P_2_) or PI(4,5)P_2_. The major phosphoinositide of the primary cilia membrane is phosphatidylinositol 4-phosphate (PI4P), whereas PI(4,5)P_2_ is limited to the ciliary base [40]. INPP5E, a cilia-localized inositol 5-phosphatase whose malfunction leads to ciliopathies and phosphoinositide compartmentalization, which in turn ensures proper protein trafficking and Hedgehog (Hh) signaling at primary cilia [41]. This study shows that PIP4K2a as a kinase functions in opposite to INPP5E to contribute to ciliopathies, which may directly link phosphoinositide signaling to primary cilium stability. In addition, PIP4K2a may also regulate ciliopathies through its interaction and phosphorylation of MIF to increase 14-3-3ζ mediated MIF nuclear translocation. Whether other kinases identified regulate the phosphorylation of MIF needs to be further investigated. In addition, disruption of primary cilia causes obesity in mice [42, 43]. However, how ciliary dysfunction leads to obesity has remained mysterious, partially because of a lack of understanding of pathways involved in the regulation of metabolism and energy homeostasis. Based on this study, an interesting hypothesis is that PIP4K2a mediated the phosphorylation of PtdIns5P to generate various phosphoinositide species may contribute to ciliopathies associated obesity, and MIF may be involved in this process. The observation that 12 months old MIF^−/−^ mice have an 18% higher body weight than age matched wildtype mice supports this hypothesis [44]. MIF may also regulate obesity through its transcriptional activity on genes directly associated metabolism and energy homeostasis, which may be a future research direction.

In addition, cytosolic MIF can also bind with different intracellular proteins to modulate their biological activities. For example, MIF can negatively regulate the activity of cytosolic jun-activation domain-binding protein (Jab1)/COP9-signalosome subunit 5 (CSN5) signaling through binding with Amino acids 50–65 and Cys 60 of MIF to regulate AP-1-dependent transcription [10, 45]. Upon damage or infection, intracellular MIF can also interact with nitrogen permease regulator-like 3 (NLRP3) to facilitate the interaction between NLRP3 and vimentin, resulting in the release of IL1β [46]. Furthermore, under ischemic or excitotoxic stress, apoptosis-inducing factor (AIF) binds with MIF and guides MIF into the nucleus to regulate DNA damage and cell death [13, 47]. These studies suggest an indirect role of MIF in the regulation of transcription and a role of nuclear MIF in the regulation of DNA damage.

In sum, we identified that MIF is located at proximal end of centrioles to control a balance between ciliary assembly and disassembly. However, MIF could also be translocated to the nucleus through PIP4k2a/14-3-3 axis to function as a transcriptional factor to regulate the expression of genes related to ciliopathies. Although our results provide novel mechanisms of how MIF regulates cilia biogenesis. However, how the balance between cytosolic/centriolar and nuclear MIF is regulated remains to be elusive.

## Materials and methods

### Cell culture and reagents

Human renal cortical tubular epithelia (RCTE) and hTERT-immortalized retinal pigment epithelial cell (hTERT-RPE-1) were maintained at 37 °C in 5% CO_2_ in DMEM/F12 containing 10% fetal bovine serum, supplemented with penicillin and streptomycin. Hela cells, mouse embryonic fibroblasts MEF (CF-1) and HEK293T cells were purchased from ATCC (Manassas, VA, USA) and cultured in DMEM containing 10% fetal bovine serum, supplemented with penicillin and streptomycin.

For plasmid transfection, Lipofectamine 3000 (Thermo Fisher) was used, according to the manufacturer’s instructions. For siRNAs transfection, Lipofectamine RNAiMAX (Invitrogene) was used, according to the manufacturer’s instructions.

Nocodazole and thymidine were purchased from Sigma. ISO-1 was purchased from EMD Millipore. Recombinant human MIF was purchased from R&D Systems. Lambda phosphatase was purchased from Santa Cruz.

### Mouse primary tubular cells isolation

After removal of renal capsules and medulla, kidney sections were minced into tiny pieces, and incubated in 10 mL of a 1% collagenase type I buffer in a 37 °C oven with gentle rotation for 30 min. Then undigested kidney tissues were removed through a 70 μm filter. The filtered cell suspension was centrifuged at 150 g for 5 min. The pellet was washed with PBS and then centrifuged and resuspended and cultured in DME/ F12 containing 2% fetal bovine serum, supplemented with penicillin and streptomycin, 10 ng/mL of epidermal growth factor, 5 μg/mL of insulin, 0.5 μg/mL of epinephrine, 36 ng/mL of hydrocortisone, 5 μg/mL of transferrin, and 4 pg/mL of triiodo-L-thyronine).

### Mice experiments

*Mif* whole-body knockout (KO) mice were fertile [48]. *Mif*^–/–^ mice were on the C57BL/6 genetic background. Kidneys were collected from 21 days and 3 months old *Mif* ^−/−^ mice and *Mif*^+/+^ mice and their littermates were used as controls. The subsequent sample processing and analysis were carried out in a blinded manner. All animal experiments were conducted under the IACUC protocol: A00003756-18, which was reviewed and approved by the IACUC of the Mayo Clinic, in accordance with the National Institutes of Health, United States Department of Agriculture, and the Association for Assessment and Accreditation of Laboratory Animal Care guidelines.

### Antibodies, drugs and reagents

The antibodies used for Western blot analysis included, (a) anti-MIF (ab36136) antibody which was purchased from Abcam; (b) anti-PIP4K2a (sc-100406), anti-14-3-3ζ (sc-293415), anti-Cyclin B (sc-4135), anti-CDC20 (sc-14866), anti-CDH1 (sc-56312), anti-GFP (sc-9996) antibodies which were purchased from Santa Cruz Biotechnology Inc; (c) anti-T/S pan (# 9631), anti-Lamin A/C (# 2032) antibodies which were purchased from Cell Signaling Technology; (d) anti-KIF3a (13930-1-AP), anti-IFT20 (13615-1-AP), anti-IFT88 (13967-1-AP), anti-IFT140 (17460-1-AP) antibodies which were purchased from Proteintech; (e) anti-β-actin antibody (A5316) antibody which was purchased from Sigma-Aldrich. The secondary antibodies, including donkey anti-rabbit IgG–horseradish peroxidase (sc-2313) and goat anti-mouse IgG–horseradish peroxidase (sc-2005), were purchased from Santa Cruz Biotechnology, Inc. The antibodies used for Immunofluorescent staining included, (a) anti-MIF (ab36136), anti-MIF (sc-20121), anti-CEP135 (ab75005), anti-CEP290 (ab84870) antibodies which were purchased from Abcam, (b) anti-GFP (sc-9996), anti-CEP164 (sc-515403), anti-Ninein (sc-376420), anti-c-Nap1 (sc-390540), anti-PIP4K2a (sc-100406) antibodies which were purchased from Santa Cruz Biotechnology Inc; (c) anti-gamma-tubulin (T5326), anti-acetylated α-tubulin (T7451), anti-TTBK2 (HPA018113) antibodies which were purchased from Sigma-Aldrich; (d) anti-CP110 (127801-AP), anti-BBS4 (12766-1-AP), anti-KIF3a (13930-1-AP), anti-IFT20 (13615-1-AP), anti-IFT88 (13967-1-AP), anti-IFT140 (17460-1-AP) antibodies which were purchased from Proteintech; (e) anti-pericentrin (ABT59) antibody which was purchased from EMD Millipore. The Alexa Fluor secondary antibody 555 and 488 were purchased from Thermo Fisher.

### DNA constructs and siRNAs

The GFP-tagged MIF (RG205106) and GFP-tagged PIP4K2a (RG205243) plasmids were purchased from Origene. The mutant MIF plasmids, including the S91D and S91A mutants were generated by KOD-Plus-Mutagenesis Kit (TOYOBO) following the manufacturer’s instructions. The RNA oligonucleotides that specifically target human MIF (sc-37137), human PIP4K2a (sc-39139), human 14-3-3ζ (sc-29583) were purchased from Santa Cruz Biotechnology, Inc.

### RNA extraction and quantitative reverse transcription-polymerase chain reaction (qRT-PCR)

Total RNA was extracted using the RNeasy Plus Mini Kit (QIAGEN). Total RNA (1 μg) was used for reverse transcription reactions in a 20 μl reaction to synthesize cDNA with an iScript cDNA Synthesis Kit (Bio-Rad). RNA expression profiles were analyzed by real-time PCR using iTaq SYBR Green Supermix with ROX (Bio-Rad) in an iCycler iQ Real-Time PCR Detection System. The complete reactions were subjected to the following program of thermal cycling: 40 cycles of 10 s at 95 °C and 20 s at 60 °C. A melting curve was run after the PCR cycles followed by a cooling step. Each sample was run in triplicate in each experiment, and each experiment was repeated three times. Expression levels of target genes were normalized to the expression level of actin. All primers used are listed in Supplementary Table 1.

### Protein extraction and western blot analysis

Cell pellets were collected and resuspended in lysis buffer (20 mM Tris-HCl, pH 7.4, 150 mM NaCl, 10% glycerol, 1% Triton X-100, 1 mM Na3VO4, 25 mM β-glycerolphosphate, 0.1 mM PMSF, Roche complete protease inhibitor set, and Sigma-Aldrich phosphatase inhibitor set). The resuspended cell pellet was vortexed for 20 s and then incubated on ice for 30 min and centrifuged at 20,000 g for 30 min. The supernatants were collected for Western blot analysis or immunoprecipitation. Full and uncropped western blots are presented in Supplemental File.

### Immunofluorescence microscopy

Briefly, cells on coverslips were fixed in pre-cold methanol for 10 min at −20 °C, followed by permeabilization with 0.1% Triton X-100 for 15 min at room temperature. After blocking in 2% BSA, cells were incubated with appropriate primary and secondary antibodies. For tissue staining, mouse kidneys were dissected out from 21 days-old or 3 months-old mice, fixed in 4% PFA in PBS at room temperature overnight, further dehydrated and infiltrated the tissue with paraffin, then embed the tissue in paraffin.

Images were acquired using a Zeiss confocal microscope LSM780 or a Nikon Eclipse 80i microscope. Three-dimensional structured illumination microscopy experiments were performed using a Zeiss ELYRA super-resolution microscopy system using 63 × 1.4 oil immersion lens. Images were processed by 3D transparency-rendering method. Cilium length marked with Ac-αTub or Arl13B was measured using the line tool in ZEN software package (Blue edition, Zeiss). An intensity profile of a yellow arrow across the ring structures was measured by ZEN software package (Blue edition, Zeiss). Other cilia protein intensity was measured by selecting a circular area of the “fluorescent dot” or the “fluorescent area” and calculating the intensity mean value by using the ImageJ. Statistical analyses were performed in GraphPad Prism version 8.0 (GraphPad Software).

### Extraction of cytoplasmic and nuclear proteins

The cytosol extracts (C) and nuclear extracts (N) are the fraction prepared from whole-cell lysates. Briefly, cell pellets were collected and resuspended in cytoplasmic extract buffer (10 mM HEPES pH 7.9, 10 mM KCl, 0.1 mM EDTA, 0.3% NP-40, protease inhibitors 1x). The resuspended cell pellet was vortexed for 5 min on ice and then centrifuged at 3000 rpm for 5 min at 4 °C. The supernatants were harvested as the cytoplasmic extracts. Resuspended and washed the pellet in cytoplasmic extract buffer without NP-40 and centrifuged at 3000 rpm for 5 min. Then resuspended the pellet in nuclear extract buffer (20 mM HEPES pH 7.9, 0.4 M NaCl, 1 mM EDTA, 25% Glycerol, protease inhibitors 1×) and incubated on ice for 10 min. Centrifuged at 14,000 rpm for 5 min at 4 °C and harvested supernatant (nuclear extract). The samples were used for Western blot analysis.

### Cell synchronization and cell cycle analyses

RCTE and hTERT-RPE-1 cells were synchronized by serum deprivation and restimulation as described [22, 27]. RCTE cells and Hela cells were also synchronized with thymidine followed by nocodazole for G2/M arrest and double thymidine for G1/S arrest.

For G2/M arrest, RCTE and Hela cells were subjected to thymidine (2 mM, Sigma-Aldrich) for 18 h, followed by release for 6 h, and subsequently blocked again with nocodazole (250 ng/mL, Sigma-Aldrich) for 12 h. For G1/S arrest, cells were subjected to thymidine (2 mM, Sigma-Aldrich) for 18 h, followed by release for 12 h, and subsequently blocked again with thymidine for 18 h. Propidium iodide staining and FACS analysis were performed as described [22]. Briefly, 70% ethanol-fixed cells were treated with 20 μg/ml RNase A and 10 μg/ml propidium iodide for 30 min before being subjected to cell cycle analyses with FACS Calibur flow cytometry (BD Biosciences).

### ChIP assay

ChIP assays were performed according to the protocol described previously [29]. Chromatin DNA was subjected to immunoprecipitation with anti-MIF, anti-H3 antibody, or normal goat IgG, and then washed, after which the DNA-protein crosslinks were reversed. Negative control primers were located about 2000 bp upstream of the transcription start site (TSS) of targeted genes. The recovered DNA was analyzed by PCR for the binding of MIF at the respective human ciliary gene promoters.

### ChIP-seq analysis

The raw data of ChIP-sequencing [13] was re-analyzed [49].

### Human protein chip high-throughput screening

Human protein chips which were prepared by spotting more than 21,000 highly purified proteins onto special nitrocellulose-coated slides, were incubated in renaturation buffer containing 50 mM Tris-HCl, pH 8.0, 100 mM NaCl, 1 mM DTT, 0.3% Tween 20 for 1 h at 4 °C. After Blocking with 5% bovine serum albumin for 1 h at room temperature, protein chips were incubated with purified human MIF protein (50 nM, R&D Systems) or 5% bovine serum albumin for 1 h. Protein interaction was then determined by sequentially incubating with either rabbit anti-MIF antibody and goat anti-rabbit immunoglobulin G (IgG)-Cy3-conjugated antibody or goat anti-rabbit immunoglobulin G (IgG)-Cy3-conjugated antibody (negative controls). Axon GenePix 4000B Microarray Scanner (Molecular Devices, LLC, Sunnyvale, CA), and the probe signals were acquired using GenePix Pro 6.0 software (Molecular Devices). The probes were considered detectable when the signal-to-noise ratios (SNRs) for both duplicates were over 1.2 as described previously [50].

### LC−MS/MS analysis

The procedure for LC-MS/MS was performed as described previously [51]. The lysates of RCTE cells were collected and immunoprecipitated with MIF antibody. Then the Immunoprecipitation samples were separated by sodium dodecyl sulfate (SDS)-polyacrylamide gel electrophoresis, and visualized with colloidal Coomassie blue staining. Protein bands visible after coomassie blue staining were subjected to in-gel reduction, carboxyamidomethylation and tryptic digestion. Digested peptides were measured on a Dionex Ultimate 3000 nano-LC system coupled to a linear quadrupole ion trap-Orbitrap mass spectrometer equipped with a nanoelectrospray ion source 16. Protein identification was performed by searching the data against the databases using Mascot Deamon. Subcellular location of the identified proteins was determined using DAVID bioinformatics resources.

### Co-immunoprecipitation (Co-IP)

For immunoprecipitation, cells were transfected with indicated plasmids and vectors, and were subjected to immunoprecipitation with anti-MIF, anti-GFP or anti-PIP4K2a antibodies coupled to protein A agarose beads (Pierce) in PBS containing 5 mg/ml bovine serum albumin (Sigma-Aldrich) for 6 h at 4 °C on a rotating platform. The cell lysates were then incubated with the beads coupled with antibodies at 4 °C overnight. Then the beads were washed with lysis buffer containing an additional 300 mM NaCl, and the immune complexes were eluted off the beads using loading buffer with a boiling for 5 min and then subjected to Western blot analysis.

### Statistical analysis

Statistical significance was determined by (multiple) student’s *t*-test or one-way ANOVA and performed using Prism v8.0 (GraphPad Software). Data were presented as mean ± SD or SEM. Differences with *p* values less than 0.05 were considered statistically significant. Experiments for quantification were performed in a blinded fashion. In order to ensure adequate power to detect the effect, at least three independent biological repeats for cell lines, and three to five independent biological repeats for mouse experiments. No samples or animals were excluded from the analysis. ns: non-significant, *p < 0.05, ***p < 0.001.

### Supplementary information

Supplementary figures, supplementary table, supplementary legends

Original Data File

aj-checklist

Data Set 1

Data Set 2

Data Set 3

Data Set 4

## Data Availability

All data needed to evaluate the conclusions in this study are presented in the paper. Additional data related to this paper may be requested from the corresponding authors.
