# Supplementary material for: Phosphorylation of MIF by PIP4K2a is necessary for cilia biogenesis
Source: Cell Death Dis. 2023 Dec 5;14(12):795. doi: 10.1038/s41419-023-06323-9 (PMC10698143; doi:10.1038/s41419-023-06323-9)
Supplement: Supplementary file 1 — Supplementary figures, supplementary table, supplementary legends [file 41419_2023_6323_MOESM1_ESM.docx]

**Supplementary information**

**
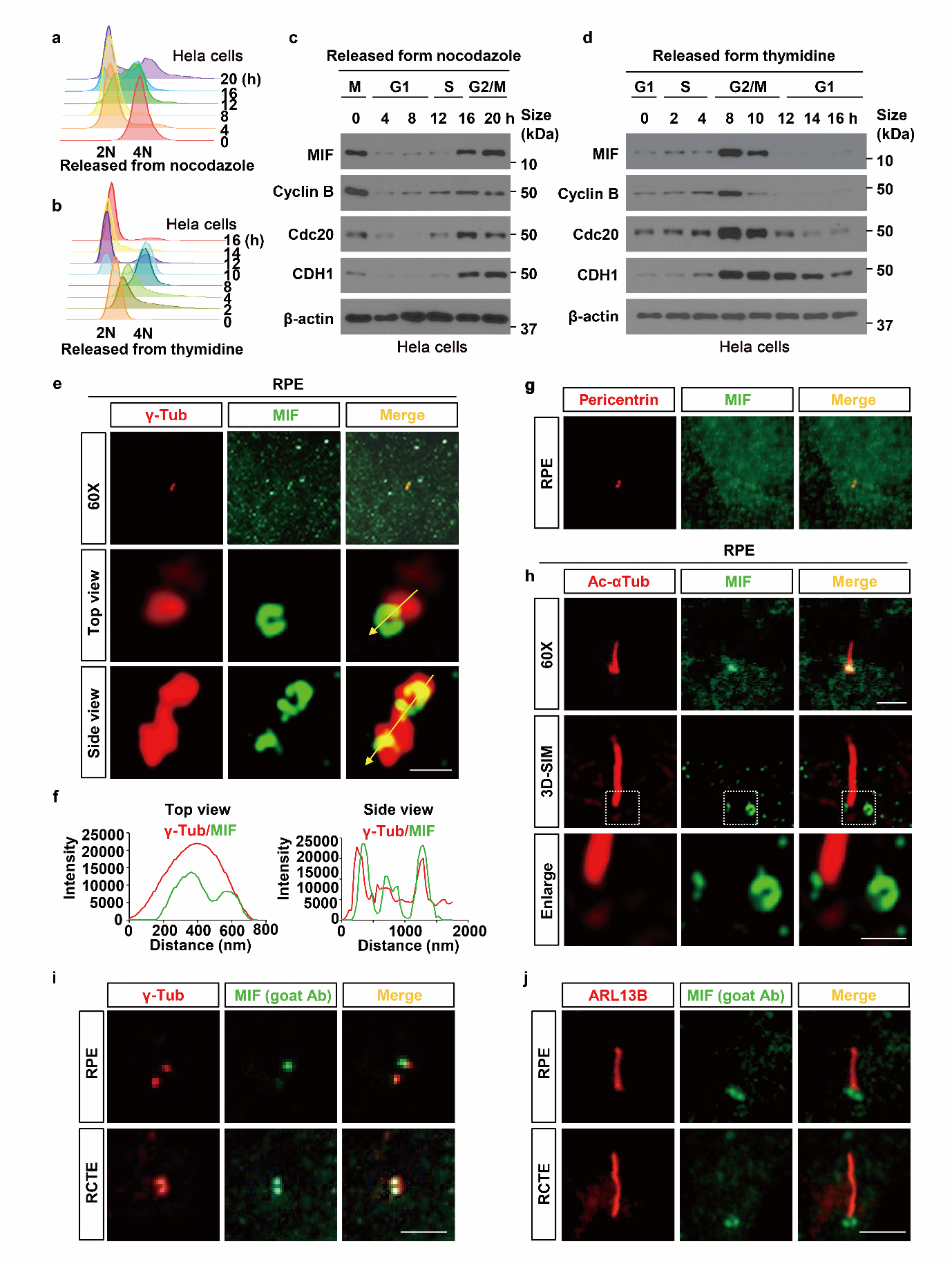
1. Supplementary Figure legends**

**Figure S1. The levels of MIF protein fluctuate throughout cell cycle and MIF is located at cilia base body.** **a**, **b** FACS analysis was used to estimate the cell-cycle profiles in thymidine-nocodazole (**a**) and double thymidine (**b**) treated Hela cells by measuring the DNA contents in those cells with propidium iodide staining. **c**, **d** Western blot analysis of whole cell lysates derived from Hela cells synchronized in M phase by thymidine-nocodazole (**c**) and in late G1/S phase by double thymidine treatment (**d**) following by releasing back into the cell cycle. **e** Representative images of RPE cells stained with MIF (green) antibody and co-stained with γ-tubulin (γ-Tub) (red), which were visualized under 2D microscope and three-dimensional structured illumination microscopy (3D-SIM). Scale bars, 5 μm (*top panels*) and 500 nm (*middle* *and bottom panels* of 3D images)**. f** The intensity plots of the rings in 3D images (**e**) from top and side views. **g** Representative images of RPE cells stained with MIF (green) antibody and co-stained with pericentrin (red). Scale bars, 5 μm. **h** Representative images of RPE cells stained with MIF (green) antibody and co-stained with acetylated α-tubulin (Ac-αTub) (red), which were visualized under 2D microscope and 3D-SIM microscopy. The top view or side view regarding the centriole structures. Scale bars, 5 μm (*top panels*) and 500 nm (*middle* *panels* of 3D images). **i**, **j** Representative images of RPE and RCTE cells stained with a second MIF antibody (green) and co-stained with γ-tubulin (red) (**i**) or ARL13b (red) antibody (**j**). Scale bars, 5 μm.


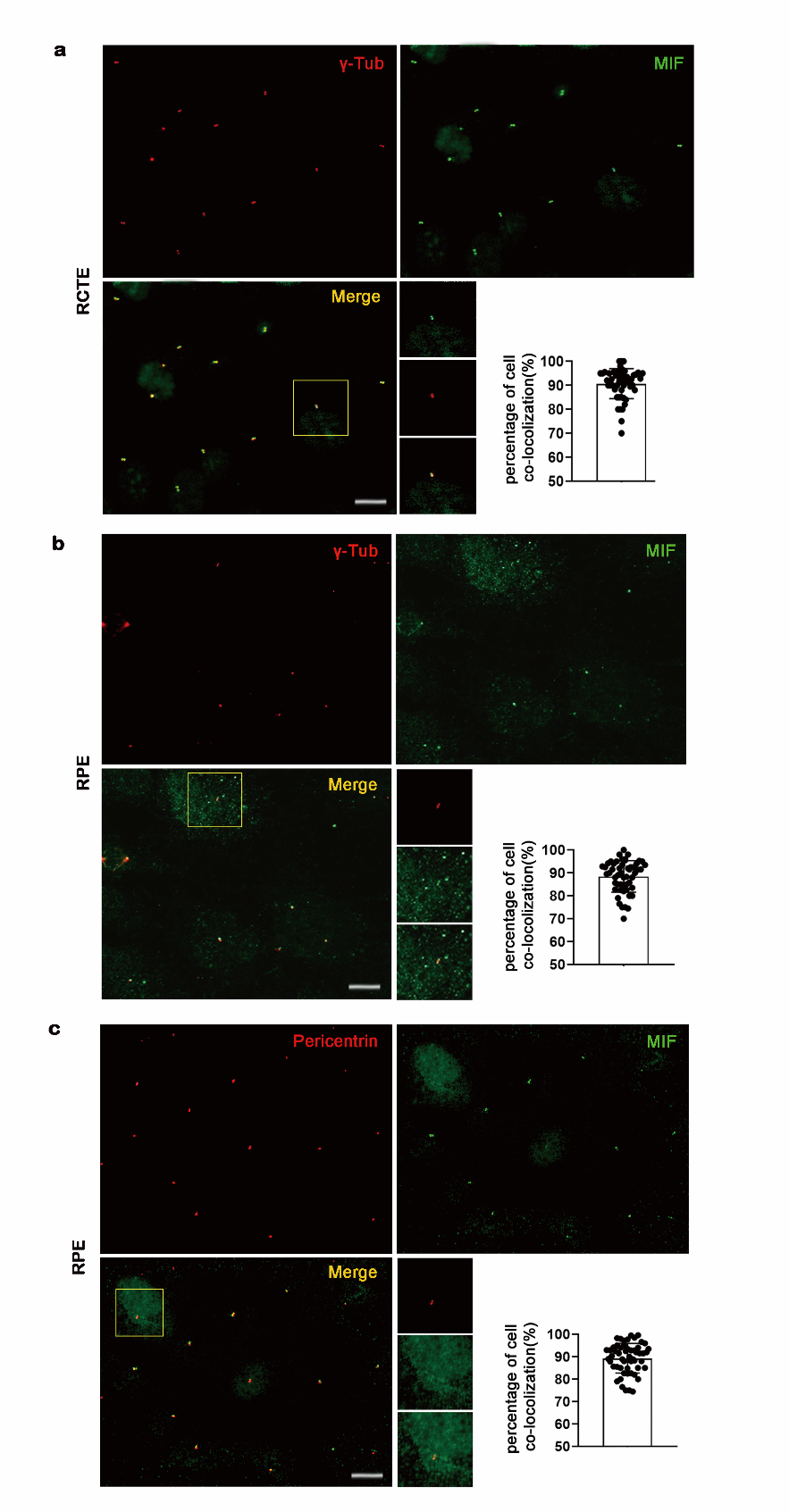


**Figure S2. MIF is co-localized with γ-tubulin at the centrioles. a** Representative image of RCTE cells stained with MIF (green) and γ-tubulin (γ-Tub) (red) antibodies. **b, c** Representative images of RPE cells stained with MIF (green) antibody and co-stained with either γ-Tub (red) (**b**) or pericentrin (red) (**c**) antibody. Scale bars, 10 μm.


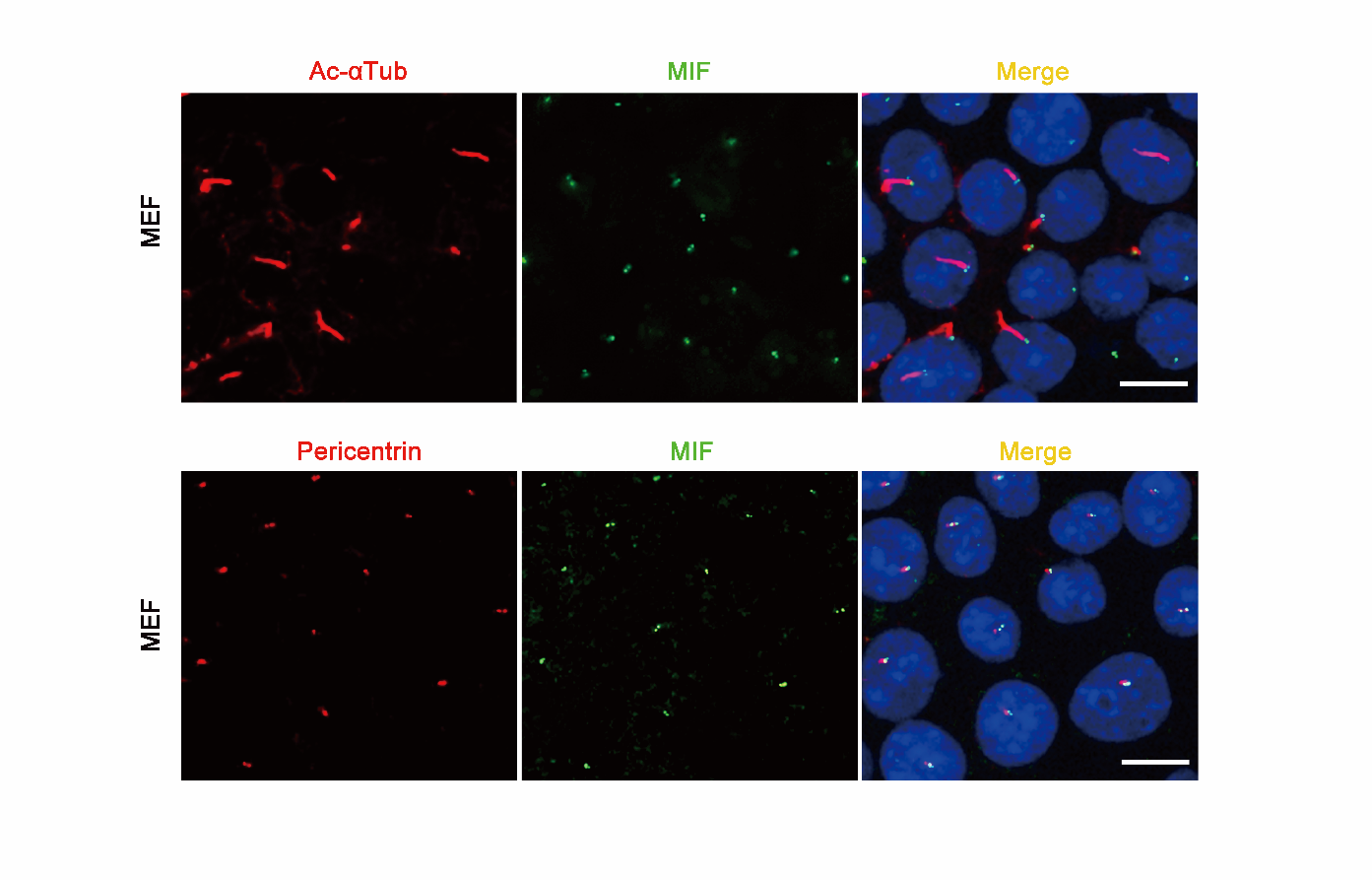


**Figure S3. MIF is located at the centrioles in MEF cells.**  Representative images of MEF cells stained with MIF antibody (green) and co-stained with either Ac-αTub (red) or pericentrin (red) antibody. Scale bars, 10 μm.


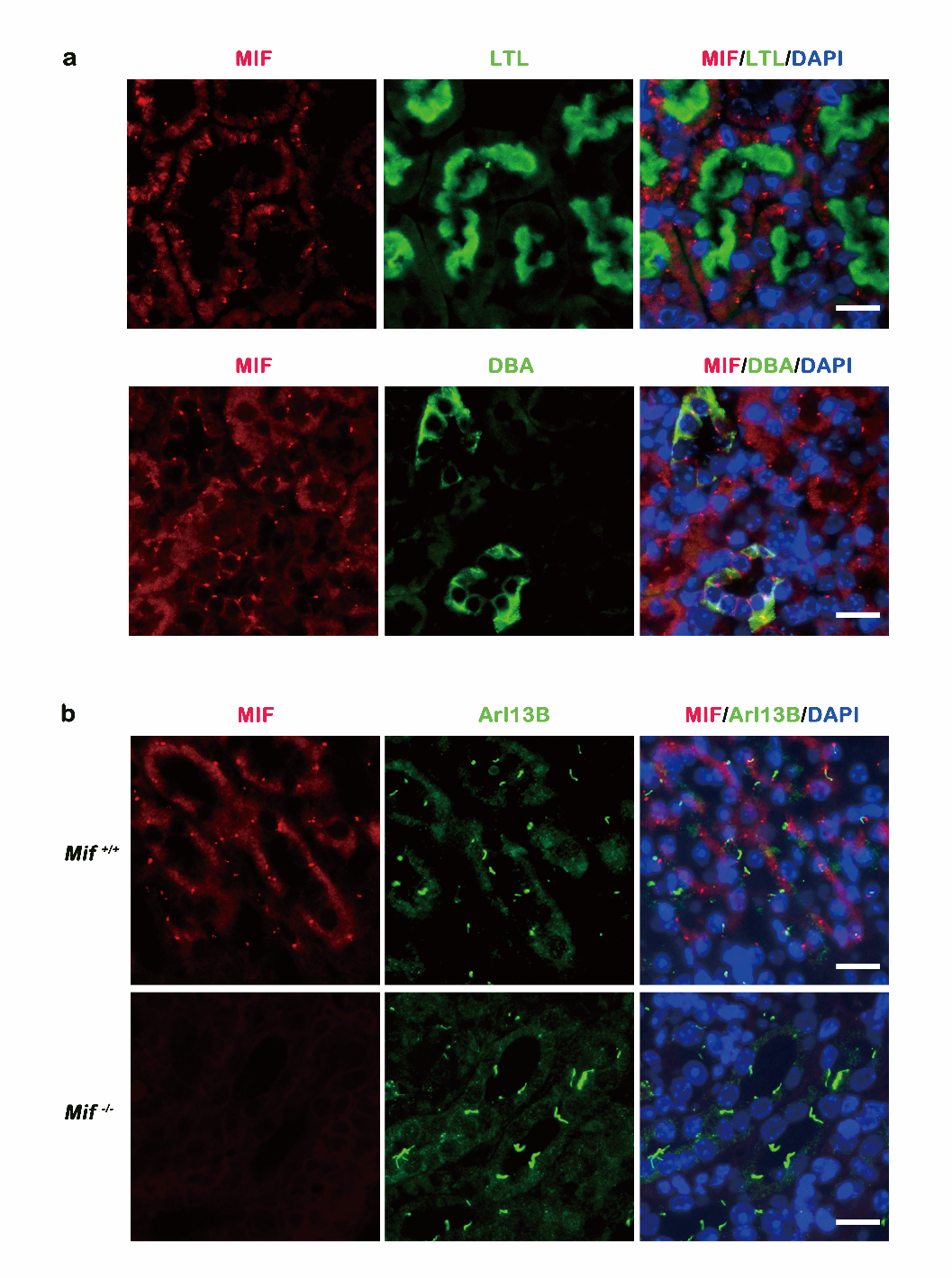
**Figure S4. MIF is mainly located at the centrioles of kidney tubular cells. a** Representative images of kidney tissue cells stained with MIF antibody (red) and co-stained with LTL (proximal tubule marker) and DBA (collecting ducts marker). **b** Representative images of kidney tissue cells stained with MIF antibody (red) and co-stained with Arl13B (green). Scale bars, 20 μm.


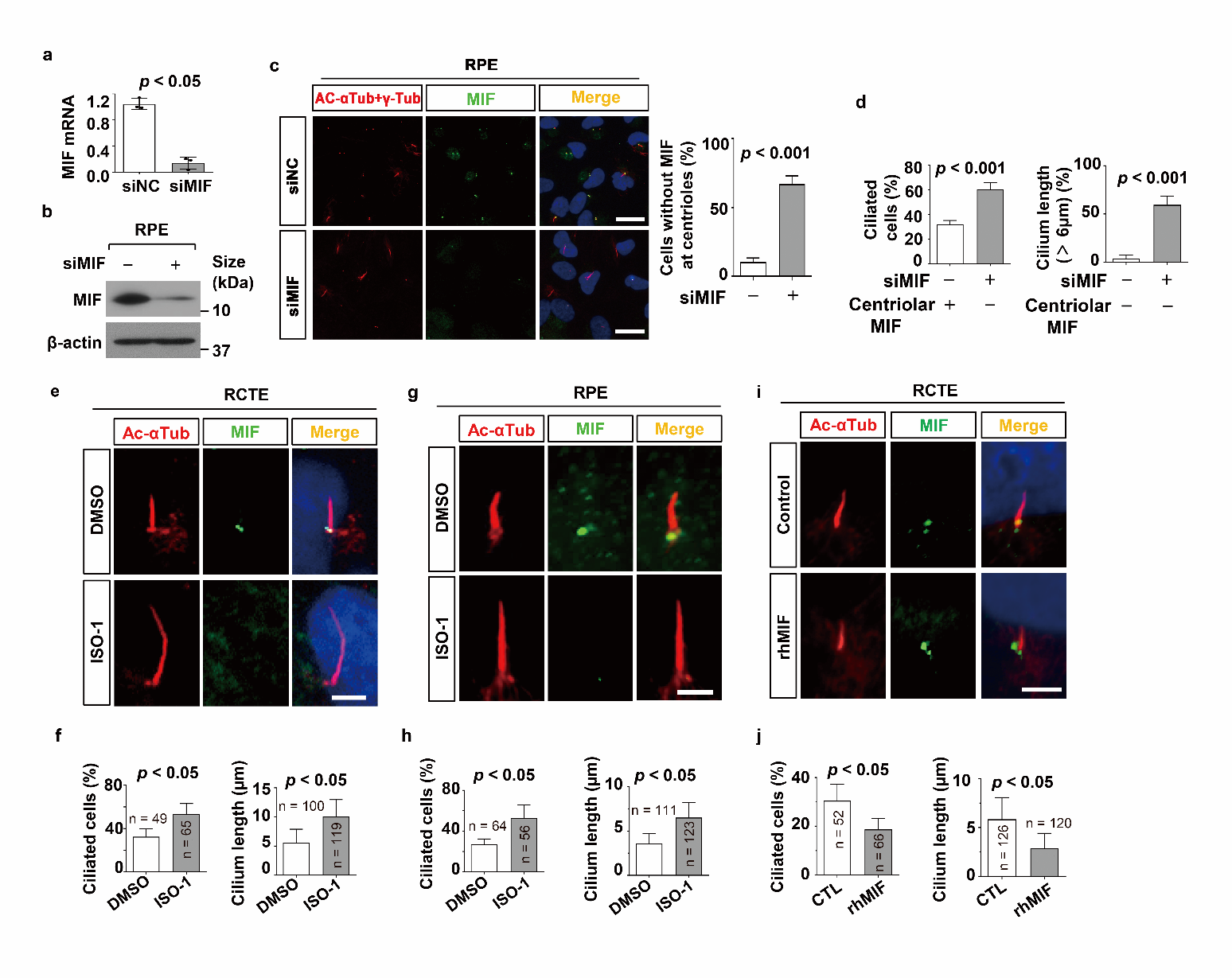
**Figure S5. Knockdown / Inhibition of MIF or treatment with rhMIF results in aberrant ciliogenesis. a-b** The levels of MIF in RPE cells transfection with MIF and control siRNA were examined with qRT-PCR (**a**) and Western blot (**b**) analysis. **c** Representative images of RPE cells transfected with MIF and control siRNAs for 48h and serum starved for 24h, and then stained with MIF (green) and Ac- αTub (red) and γ-Tub (red) antibodies. Scale bars, 20 μm. We quantified the percentage of cells without MIF at the centrioles in over 100 cells in indicated group, about 68% of cells were missing MIF at centrioles in MIF siRNA treated cells, whereas about 11% of control siRNA treated cells had MIF at centrioles. **d** Knockdown of MIF in RPE cells (**c**) increased the percentage of ciliated cells and cilia length. We quantified the percentage of ciliated cells and cilia length (＞ 6 μm) in cells with missing MIF at centrioles. For each group, over 100 cells were counted. **e**-**h** Representative images of RCTE cells (**e**) or RPE cells (**g**) treated with DMSO or ISO-1 (100 μM) plus serum free for 24h, and then stained with MIF and Ac-αTub (red) antibodies. Scale bars, 5 μm. **f**, **h** Inhibition of MIF in RCTE cells (**e**) or RPE cells (**g**) increased the percentage of ciliated cells (left) and cilia length (right). **i,** Representative image of RCTE cells treated with rhMIF (10 ng/ml) and vehicle (control) plus serum free for 24h, and then stained with MIF (green) and Ac-αTub (red) antibodies and co-stained with DAPI (blue). Scale bars, 5 μm. **j** Treatment with rhMIF in RCTE cells (**i**) decreased the percentage of ciliated cells (left) and cilia length (right). For percentage of ciliated cells, we quantified the cells in microscopic fields of view; for cilia lengths, we counted over 100 cells in each group. Numbers of high-power field views (left graph) and cells (right graph) analyzed are indicated above each dataset or in the bars, respectively, for each group (**f,h,j**). Statistical analysis was performed by unpaired two-tailed Student’s t-test. *p* < 0.05.

**
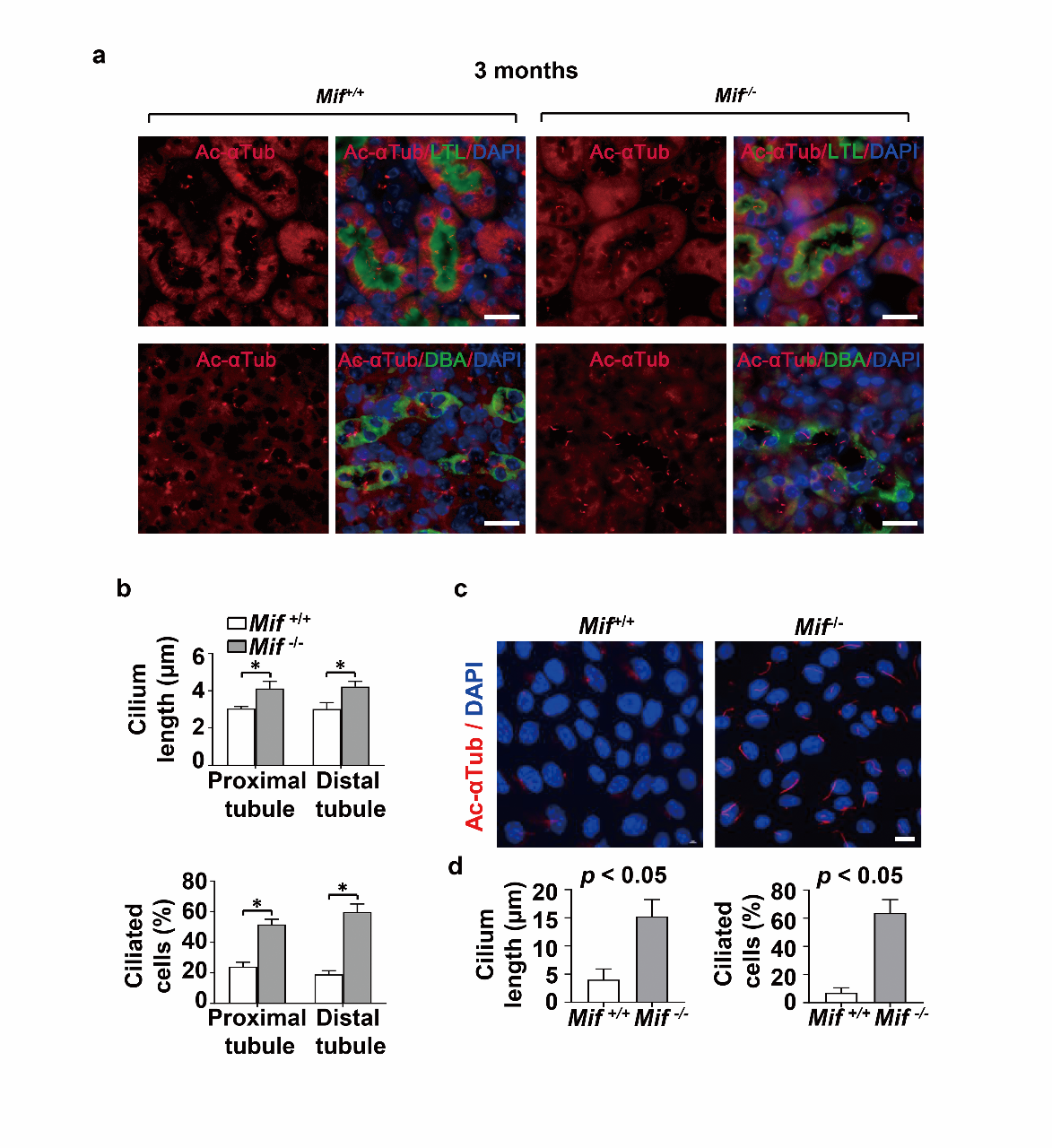
Figure S6. Knockout of MIF facilitated ciliogenesis in primary renal tubule cells and in the lumens of renal tubules.** **a** Representative images of kidney sections from 3 months old (**c**) *Mif ^+/+^* and *Mif ^–/–^* mice (n = 5 mice for each group) stained with Ac-αTub (red), the proximal tubule marker LTL (Lotus tetragonolobus lectin), the collecting duct marker DBA (Dolichos biflorus agglutinin) and DAPI (blue). White dashed lines represent the border of each renal tubule. Scale bars, 25 μm. **b** Knockout of Mif in kidneys (**a**) increased cilia length (top) and the percentage of ciliated cells (bottom) in the lumens of renal tubules compared to the controls, about 50 proximal tubule or distal tubule/collecting duct cells were measured. **c** Representative images of primary renal tubule cells isolated form 3 months old *Mif ^+/+^* and *Mif ^–/–^* kidneys (n = 5 mice for each group) and stained with Ac-αTub (red) and DAPI (blue). Scale bars, 10 μm. **d** Knockout of Mif in primary renal tubular cells (**c**) increased cilia length (left) and the percentage of ciliated cells (right) compared to the controls. Data are represented as the mean value ± s.d for three independent experiments. Statistical analysis was performed by unpaired two-tailed Student’s t-test. *p* < 0.05.

**
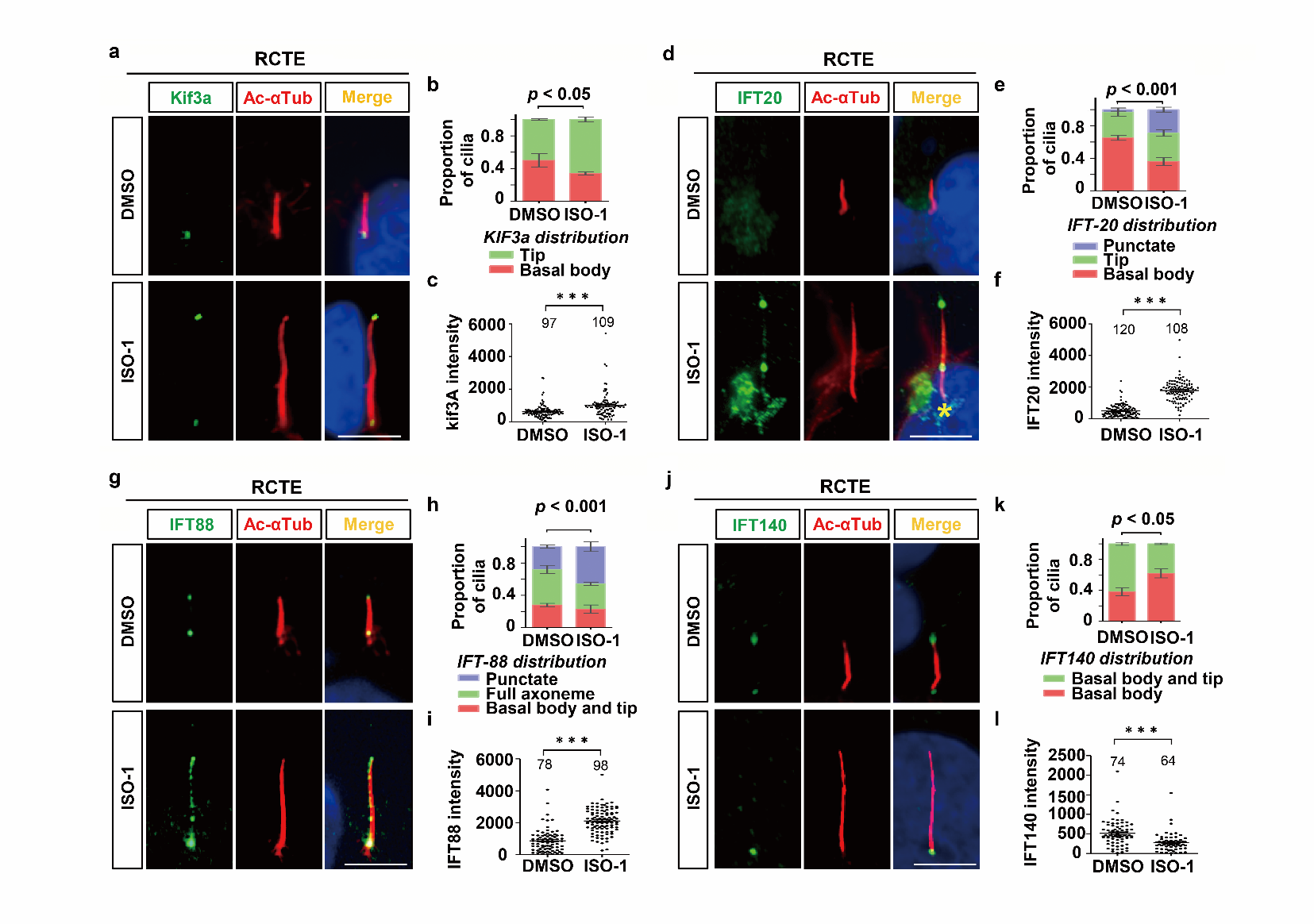
Figure S7. Inhibition of MIF affects the trafficking of KIF3a and IFT particles, including IFT20, IFT88 and IFT140.** **a** Representative image of RCTE cells treated with ISO-1 and DMSO, then serum starved for 24h and stained with KIF3a (green) and Ac-αTub (red) antibodies and co-stained with DAPI (blue). Scale bars, 5 μm. **b** Stacked bar graph represented KIF3a distribution at primary cilia in RCTE cells treated with ISO-1 and DMSO. **c** Quantification of the intensity of KIF3a signals in RCTE cells treated with ISO-1 and DMSO. **d** Representative images of RCTE cells treated with ISO-1 and DMSO, then serum starved for 24h and stained with IFT20 (green) and Ac-αTub (red) antibodies and co-stained with DAPI (blue). Scale bars, 5 μm. **e** Stacked bar graph represented IFT20 distribution at primary cilia in RCTE cells treated with ISO-1 and DMSO. **f** Quantification of the intensity of IFT20 signals in RCTE cells treated with ISO-1 and DMSO. **g** Representative images of RCTE cells treated with ISO-1 and DMSO, then serum starved for 24h and stained with IFT88 (green) and Ac-αTub (red) antibodies and co-stained with DAPI (blue). Scale bars, 5 μm. **h** Stacked bar graph represented IFT88 distribution at primary cilia in RCTE cells treated with ISO-1 and DMSO. **i** Quantification of the intensity of IFT88 signals in RCTE cells treated with ISO-1 and DMSO. **j** Representative images of RCTE cells treated with ISO-1 and DMSO, then serum starved for 24h and stained with IFT140 (green) and Ac-αTub (red) antibodies and co-stained with DAPI (blue). Scale bars, 5 μm. **k** Stacked bar graph represented IFT140 distribution at primary cilia in RCTE cells treated with ISO-1 and DMSO. **l** Quantification of the intensity of IFT140 signals in RCTE cells treated with ISO-1 and DMSO. Numbers of cells analyzed were indicated above each dataset for each group. Statistics were performed using one-sided one-way ANOVA followed by post hoc LSD test (**b**, **e**, **h**, **k**). Statistical analysis was performed by student’s *t*-test (**c**, **f**, **i**, **l**), ****p* <0.001, *****p* <0.0001.

**
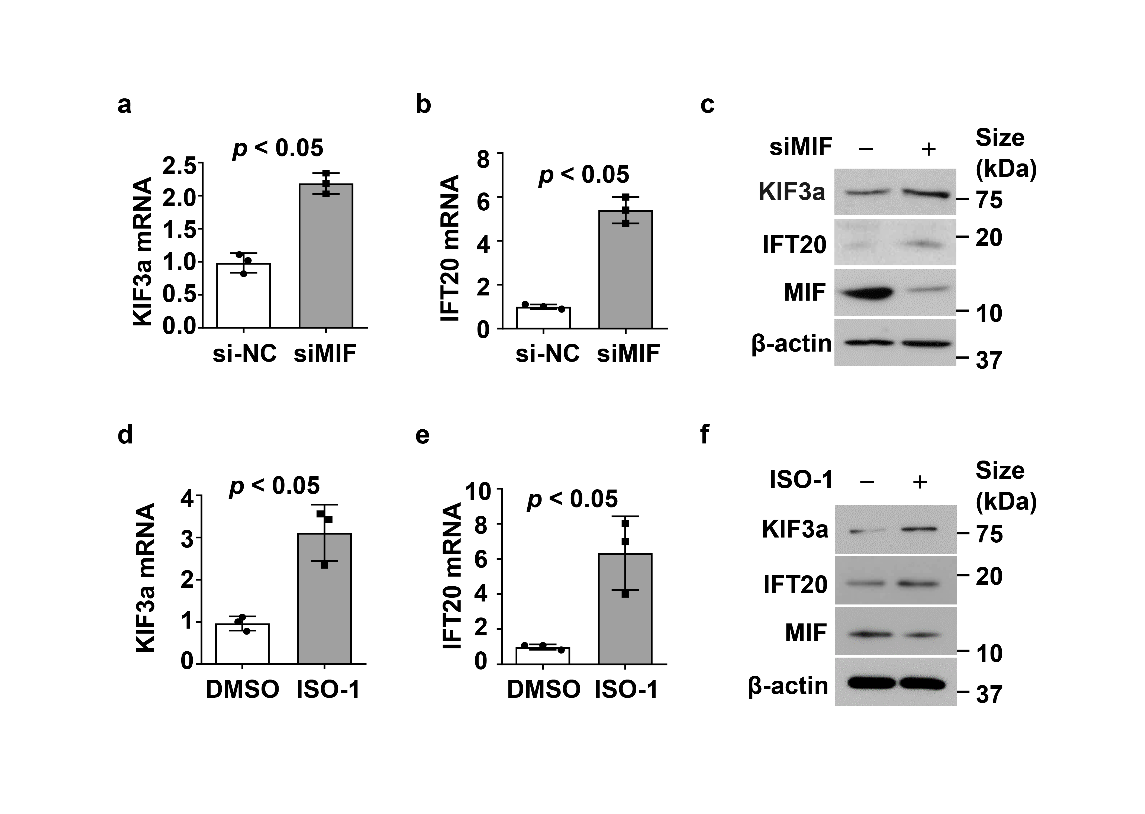
**

**Figure S8**. **Knockdown and inhibition of MIF increased the mRNA and protein levels of KIF3a and IFT20 in RCTE cells.**  **a-c** Knockdown of MIF with siRNA increased the expression of KIF3a and IFT20 in RCTE cells compared to that in the control cells as examined with RT-PCR (**a** and **b**) and Western blot (**c**) analysis. **d-f** Inhibition of MIF with ISO-1 increased the expression of KIF3a and IFT20 in RCTE cells compared to that in the control cells as examined with RT-PCR (**d** and **e**) and Western blot (**f**) analysis. *n* = 3 biologically independent experiments. All data are represented as the mean value ± s.d. Significant differences were identified by student’s *t*-test.


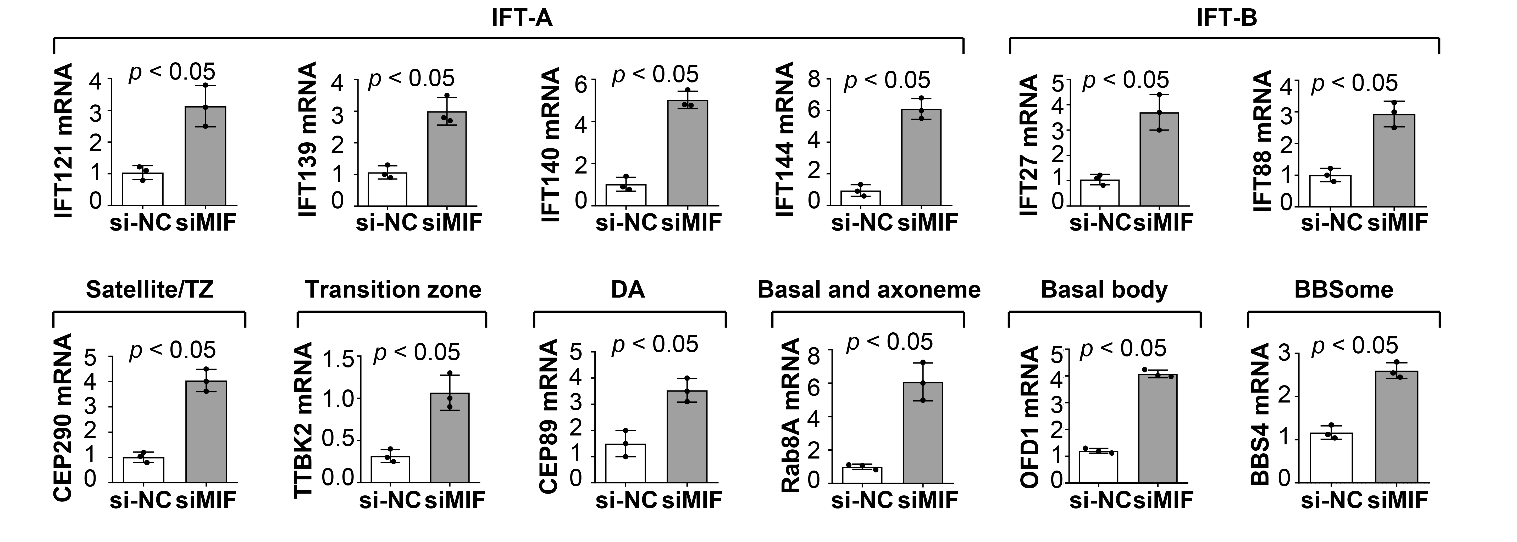


**Figure S9**. **MIF regulates the transcription of ciliary genes.** qRT-PCR analysis of the expression of ciliary genes, including IFT A particles (IFT121, IFT139, IFT140, IFT144), IFT B particles (IFT27, IFT88), centriolar satellite/ transition zone protein (CEP290), transition zone protein (TTBK2), distal appendage protein (CEP89), basal body and axoneme protein (Rab8a), basal body protein (OFD1) and BBSome protein (BBS4) in RCTE cells transfected with MIF and control siRNAs. n = 3 biologically independent experiments. All data are represented as the mean value ± s.d. Significant differences were identified by student’s *t*-test.


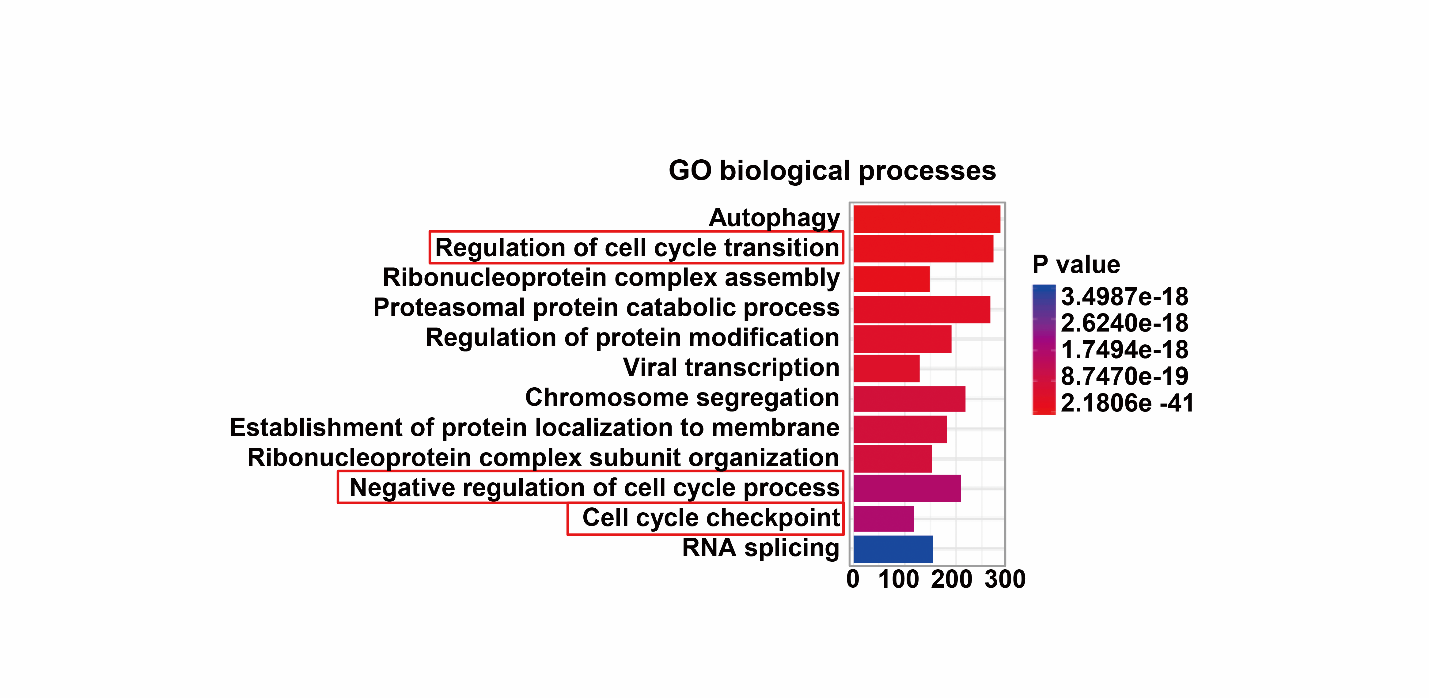


**Figure S10.** **GO biological processes analysis of MIF target genes.** The analysis of biological processes categories identified potential MIF target genes related to signaling pathways of autophagy, cell cycle transition and cell cycle checkpoint, etc.


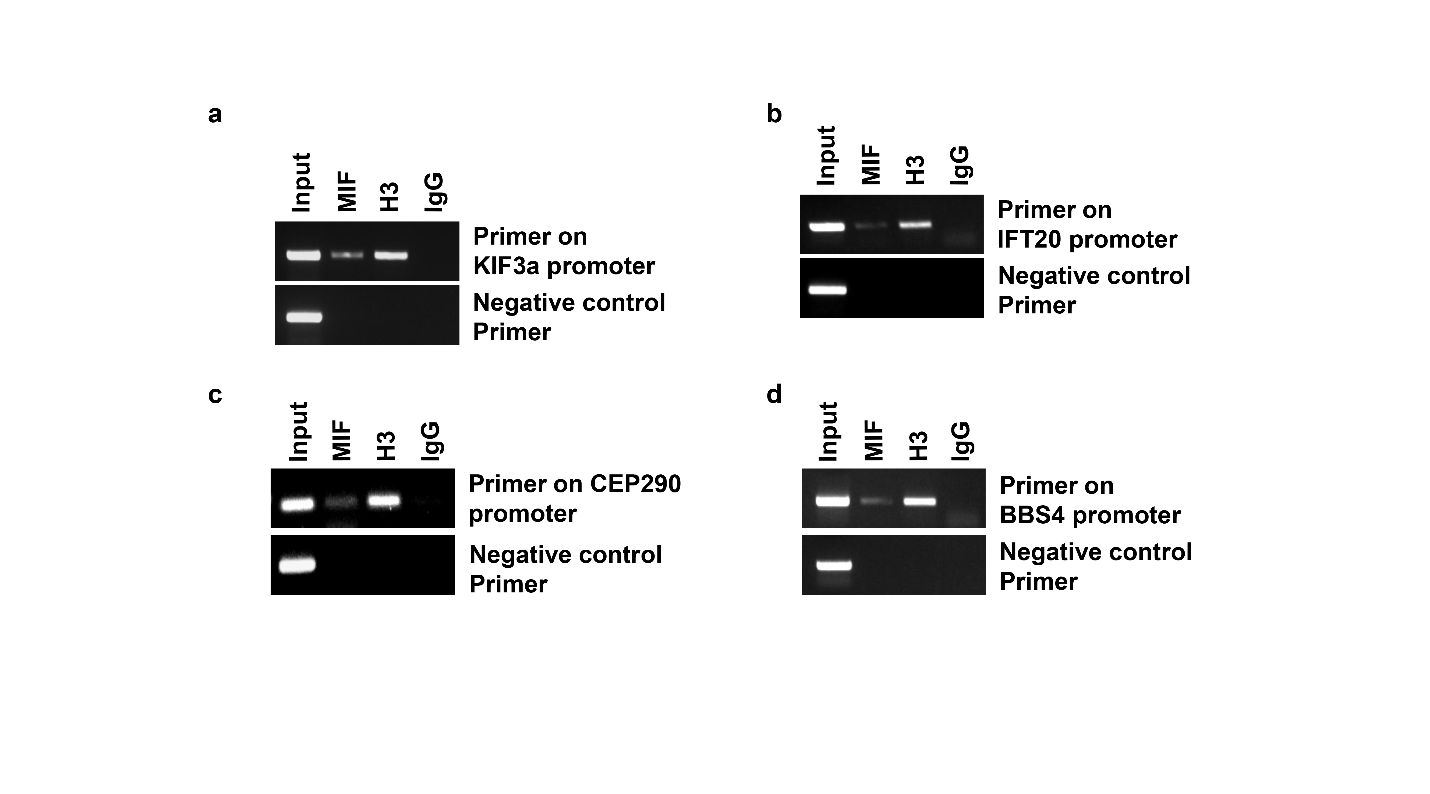


**Figure S11.** **MIF directly binds to the promoter of ciliary genes, including *IFT20, KIF3a, CEP290* and *BBS4*.** **a**-**d** ChIP assay was performed with anti-MIF antibody or IgG (control) to detect the binding of MIF on the promoters of *KIF3a* (**a**), *IFT20* (**b**)*, CEP290* (**c**) and *BBS4* (**d**) in RCTE cells. Anti-H3 antibody was used as a positive control. Negative control (NC) primers were located about 2000 bp upstream of the transcription start site (TSS) of tested genes.

**
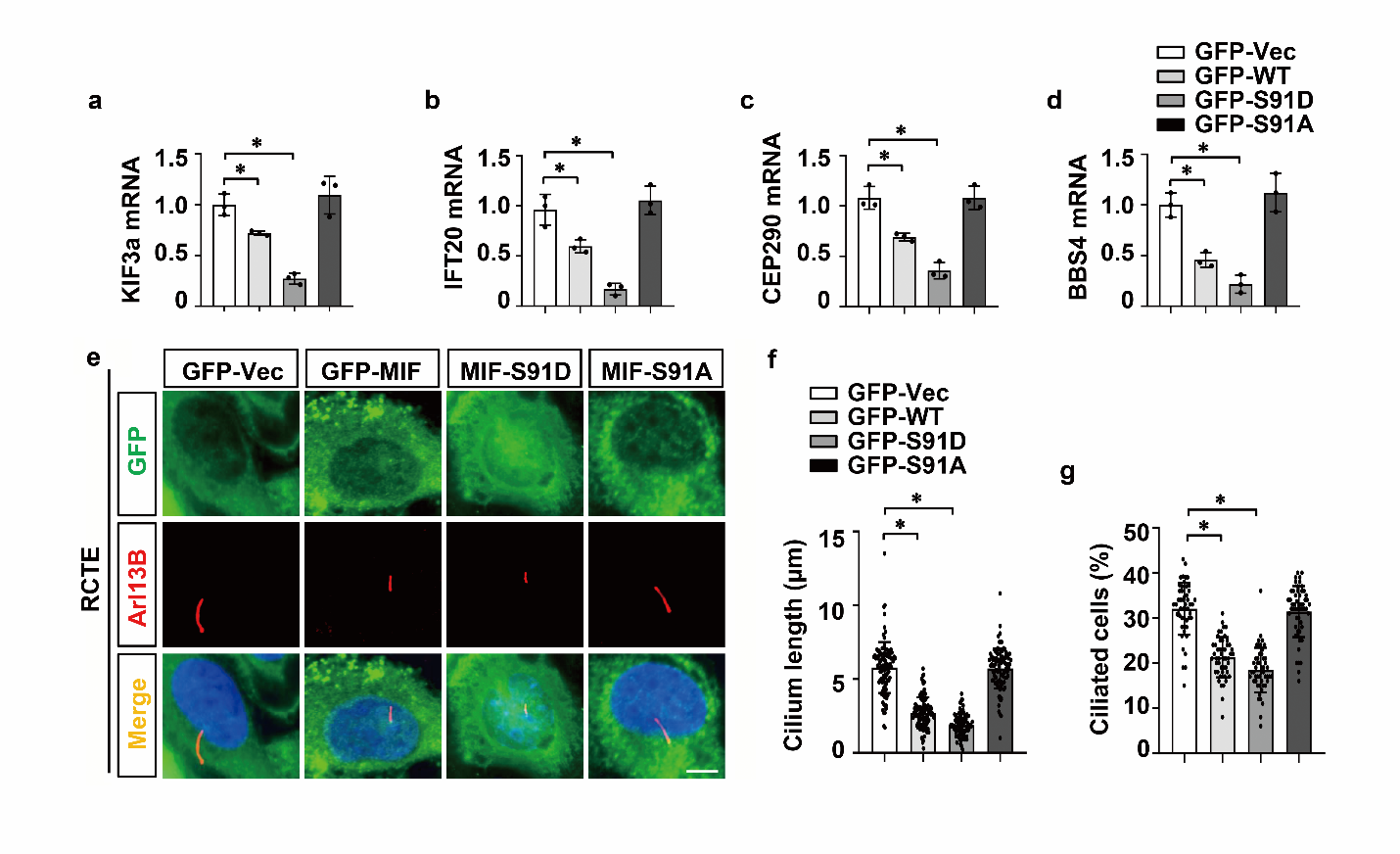
**

**Figure S12**. **Overexpression of GFP tagged wild type MIF and mutant MIF-S91D decreased the expression of cilia related genes and** **ciliogenesis in RCTE cells. a**-**d** qRT-PCR analysis of the expression of KIF3a (**a)**, IFT20 (**b**), CEP290 (**c**) and BBS4 (**d**) in RCTE cells transfected with GFP-Vector, GFP tagged wild type MIF, GFP tagged mutant MIF-S91D and MIF-S91A plasmids, respectively. *n* = 3 biologically independent experiments. Data are represented as the mean value ± s.d. Significant differences were identified by student’s *t*-test. **p* <0.05. **e** Representative images of RCTE cells transfected with GFP-Vector, GFP tagged wild type MIF, GFP tagged MIFS91D or GFP-MIF-S91A plasmids, respectively, and stained with GFP (green) and ARL13b (red) antibodies and co-stained with DAPI (blue). Scale bars, 5 μm. **f**, **g** Statistical analysis of cilium length (**f**) and the percentage of ciliated cells (**g**) in each group of RCTE cells from (**e**). Cilia length (n = 50 views for each group) and the percentage of ciliated cells (n = 100 for each group) were measured and statistically analyzed by student’s *t*-test. **p* <0.05.

**
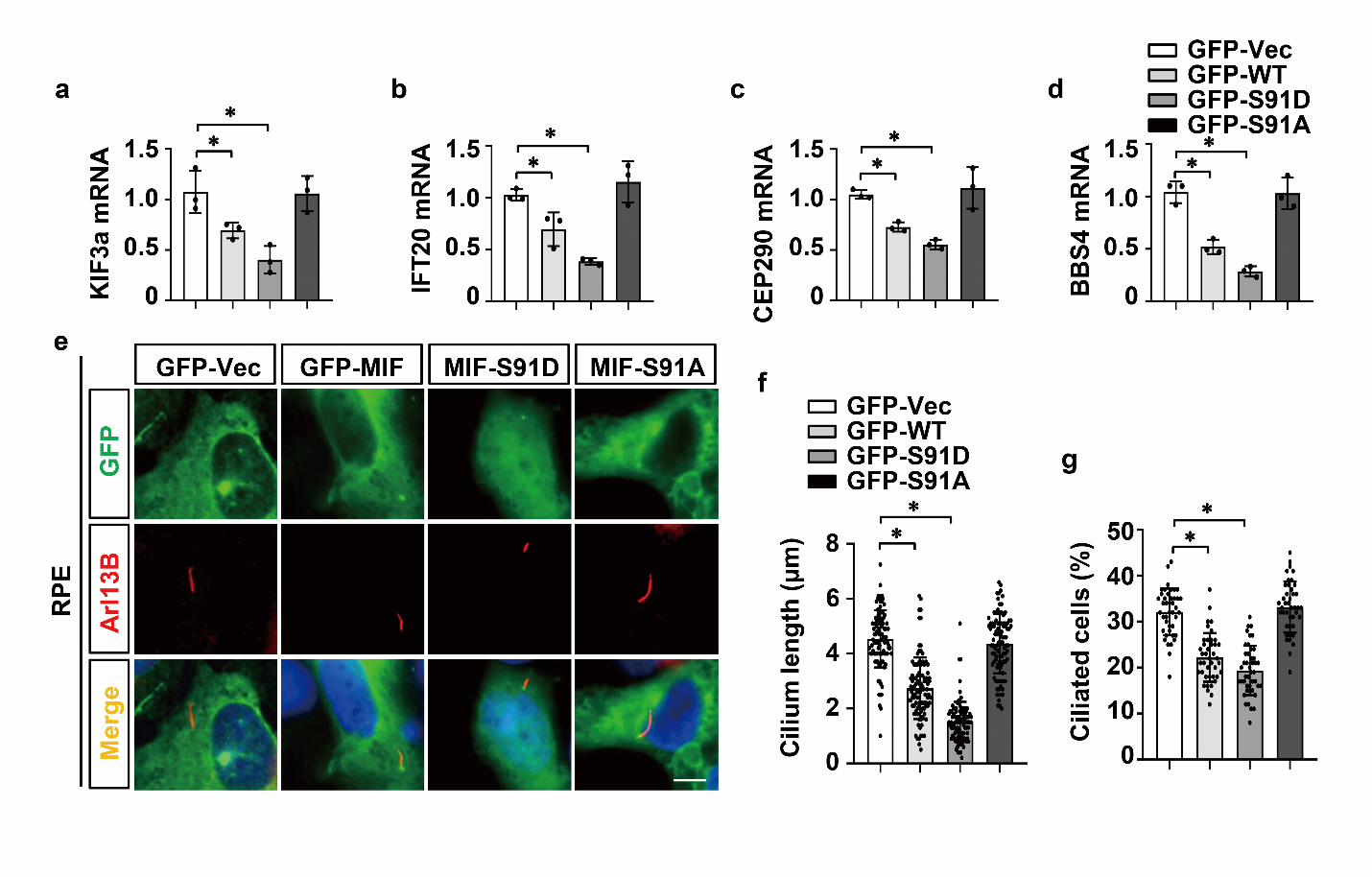
**

**Figure S13. Overexpression of GFP tagged wild type MIF and mutant MIF-S91D decreased the expression of cilia related genes and** **ciliogenesis in RPE cells. a**-**d** qRT-PCR analysis of the expression of KIF3a (**a**), IFT20 (**b**), CEP290 (**c**) and BBS4 (**d**) in RPE cells transfected with GFP-Vector, GFP tagged wild type MIF, GFP tagged mutant MIF-S91D and MIF-S91A plasmids, respectively. *n* = 3 biologically independent experiments. Data are represented as the mean value ± s.d. Significant differences were identified by student’s *t*-test. **p* <0.05. **e** Representative images of RPE cells transfected with GFP-Vector, GFP tagged wild type MIF, GFP tagged MIFS91D or GFP-MIF-S91A plasmids, respectively, and stained with GFP (green) and ARL13b (red) antibodies and co-stained with DAPI (blue). Scale bars, 5 μm. **f**, **g** Statistical analysis of cilium length (**f**) and the percentage of ciliated cells (**g**) in each group of RPE cells from (**e**). *n* = 3 biologically independent experiments. Cilia length (n = 50 views for each group) and the percentage of ciliated cells (n = 100 for each group) were measured and statistically analyzed by student’s *t*-test. **p* <0.05.

**
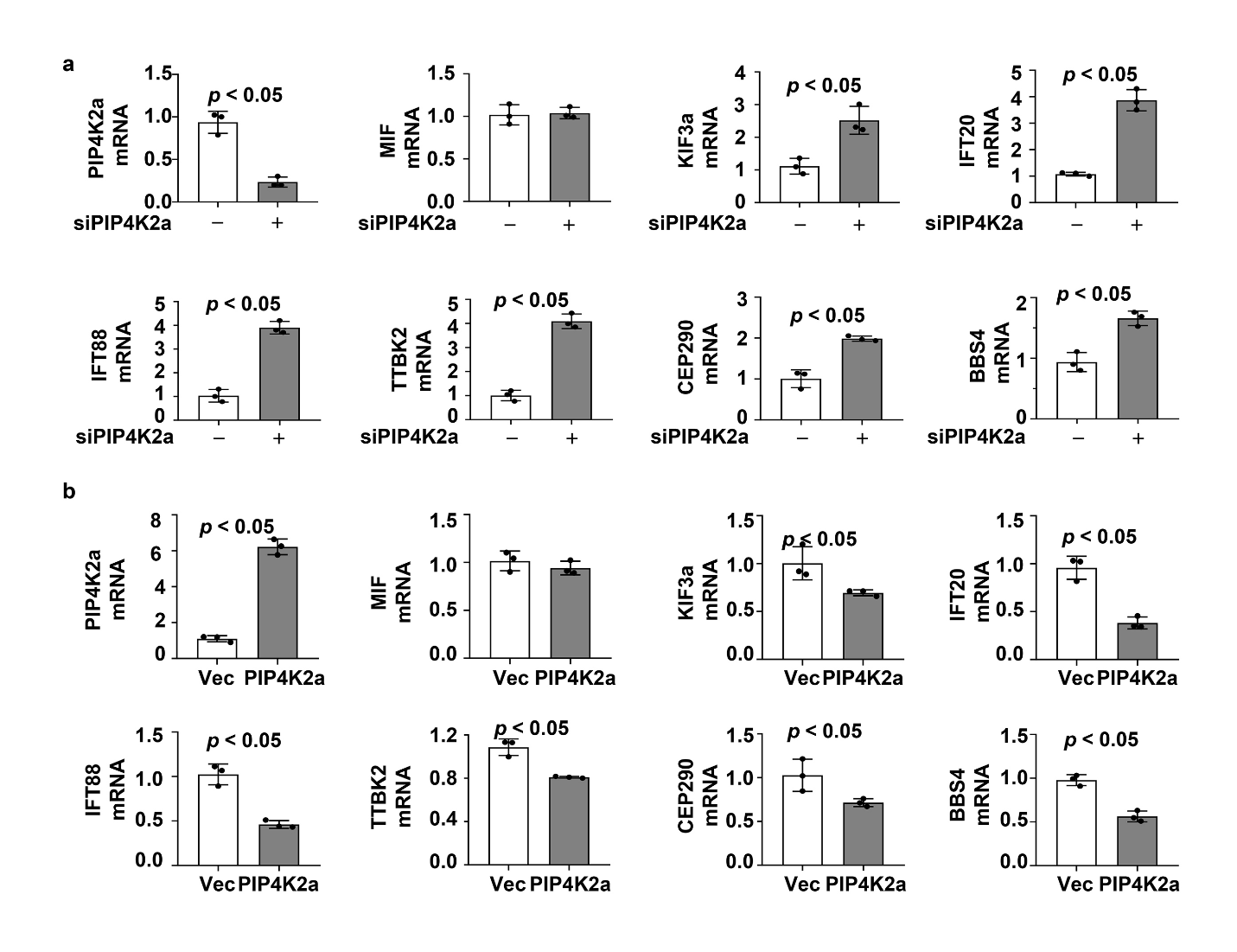
**

**Figure S14. Knockdown or overexpression of PIP4K2a affects the transcription of ciliary genes in RCTE cells. a** qRT-PCR assay was performed to detect the expression of PIP4K2a, MIF, KIF3a, IFT20, IFT88, TTBK2, CEP290 and BBS4 in RCTE cells transfected with PIP4K2a and control siRNAs. **b** qRT-PCR assay was performed to detect the expression of PIP4K2a, MIF, KIF3a, IFT20, IFT88, TTBK2, CEP290 and BBS4 in RCTE cells transfected with GFP-vector and GFP tagged PIP4K2a plasmids. *n* = 3 biologically independent experiments. All data are represented as the mean value ± s.d. Significant differences were identified by student’s *t*-test. **p* <0.05.

**
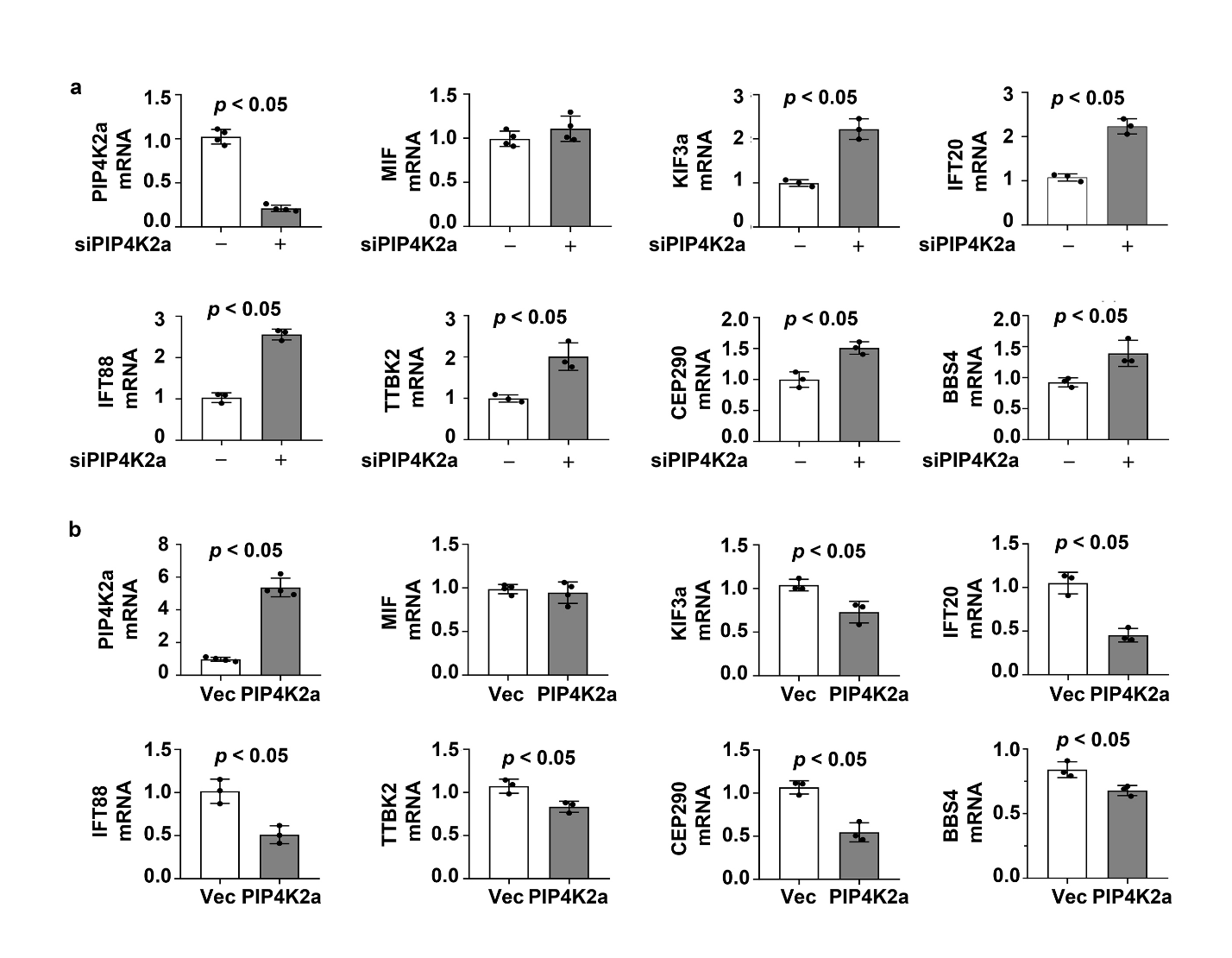
**

**Figure S15. Knockdown or overexpression of PIP4K2a affects the transcription of ciliary genes in RPE cells.**  **a** qRT-PCR assay was performed to detect the expression of PIP4K2a, MIF, KIF3a, IFT20, IFT88, TTBK2, CEP290 and BBS4 in RPE cells transfected with PIP4K2a and control siRNAs. **b** qRT-PCR assay was performed to detect the expression of PIP4K2a, MIF, KIF3a, IFT20, IFT88, TTBK2, CEP290 and BBS4 in RPE cells transfected with GFP-vector and GFP tagged PIP4K2a plasmids. *n* = 3 or 4 biologically independent experiments. All data are represented as the mean value ± s.d. Significant differences were identified by student’s *t*-test. **p* <0.05.

**2. Supplementary Tables**

**Table S1. Primers used for quantitative real time PCR and ChIP assay.**

| **Gene name** | **Forward (5’- 3’)** | **Reverse (5’- 3’)** |
| --- | --- | --- |
| ***MIF*** | AGCAGCTGGCGCAGGCCAC | CTCGCTGGAGCCGCCGAAGG |
| ***IFT20*** | AGGCAGGGCTGCATTTTGAT | CAAGCTCAATTAGACCACCAACA |
| ***IFT88*** | CAGTGACAGTGGCCAGAACA | ATCTGTCCCAGGCAAAGCTC |
| ***KIF3a*** | AAAGCGGAGGGTGATACAAG | AACTCCCACATCAGGTCTTTC |
| ***BBS4*** | AAGCAGTGGAGTTCTCACCA | GGCCTTGTAGTTGGTAGGGT |
| ***Rab8a*** | ACATCACCAACGAGAAGTCC | TTTTCTCCCCGTTCCTTGG |
| ***IFT121*** | CATTCAAGGCACAAGGGATC | GGTAGGCTCGACAATTAAGGG |
| ***IFT122*** | CAGTGCCTCGATATAGCTCAAG | AACGCTGGAAGTGGTAGAAC |
| ***IFT27*** | TTCCAGAAAAGCTACACCCTG | ACTCTCCCACAATTTATCCAGC |
| ***TTBK2*** | AGATTTATTGGCTGTGGGAGG | GACTCCAAAATCTGTCTACCCAG |
| ***IFT139*** | GAGACAAAGGGTGGGAGTGA | ACCCTCCAGTAACCTCATGC |
| ***IFT140*** | ATGGACACAGTGACCGTCTT | TCTGGTCCCAGAAGTGGTTC |
| ***IFT144*** | CACTTGTGGATGTTGGAATGC | CATGTTGCTAGGCTCTGATCTC |
| ***CEP290*** | AGACAAATGGCCTGGGATCA | GGCAAACTAGGGTCAGGGAT |
| ***CEP89*** | GTGCCAAATGCCAAGAACTC | GTCAGCTTCTCCATCAACTCC |
| ***OFD1*** | GGGATATGTCTCATGTGGACG | TCTCTGCCTTCGTTCTTTCAC |
| ***GAPDH*** | TGCACCACCAACTGCTTAGC | GGCATGGACTGTGGTCATGAG |
| ***CEP290 promoter*** | GACTGCTCCCCTCAATTCC | TTTTCCTAAGCCATCCCCTG |
| ***CEP290 promoter-NC*** | AAAGTCTGTCCATATATTTTAGGTTGAG | TTTGATTCCTGATCCTTACTCTGG |
| ***IFT20 promoter*** | AGTTTCCACTGATAGCCACAG | CATTAGCTTCGTTGCGTGAG |
| ***IFT20 promoter-NC*** | CCTCCGCTACCTCTCATAAAAC | GATGTTGGTCTTCCCTGTCC |
| ***KIF3a promoter*** | AAATCCCACTCTGTGCCAG | CTACCTGACCTCGCTTGATTG |
| ***KIF3a promoter-NC*** | CTCCTAACCTCAAGTGTTCCTC | GAATTTGCAACAGGGTGTGG |
| ***BBS4 promoter*** | CAGACAGGTAATGGAGAACGC | AATCGGCCATGGGATAAAGAG |
| ***BBS4 promoter-NC*** | AACACTTTGGGAGGTCAAGG | GTTTTGCCATATTGCCCAGG |

**3. Supplementary Data set.**

**Data Set 1.** The potential MIF target genes related to cilia biogenesis identified with ChIP-seq.

**Data Set 2.** The potential MIF target genes other than those related to cilia biogenesis.

**Data Set 3.** The potential MIF binding proteins identified by protein chip analysis.

**Data Set 4.** The potential MIF binding proteins identified by immunoprecipitation and mass spectrometry (MS) analysis in RCTE cells.
