# Supplementary material for: Phosphorylation of MIF by PIP4K2a is necessary for cilia biogenesis
Source: Cell Death Dis. 2023 Dec 5;14(12):795. doi: 10.1038/s41419-023-06323-9 (PMC10698143; doi:10.1038/s41419-023-06323-9)

Uncropped western blots

Source Data- western blots-Fig.1e

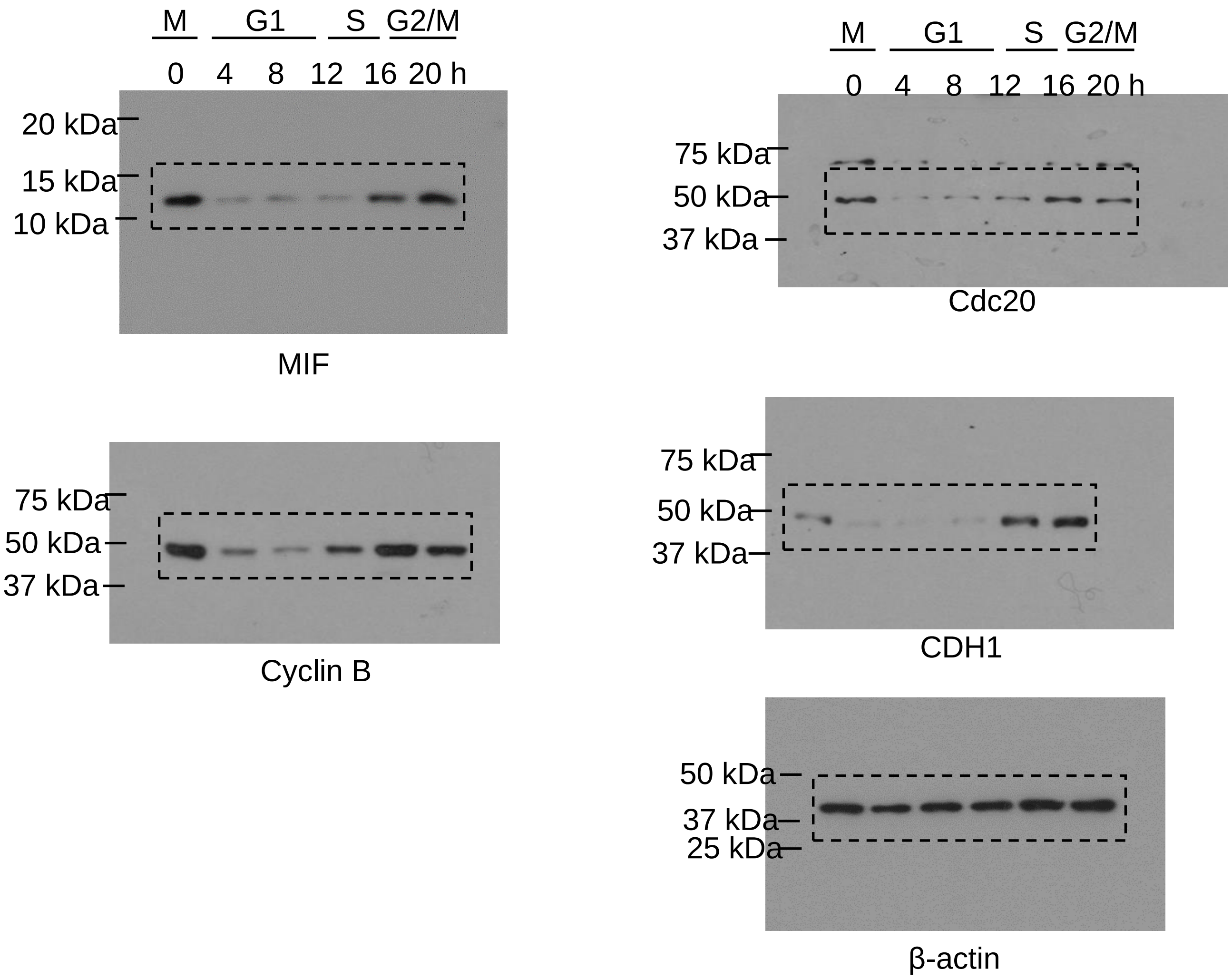

Source Data- western blots-Fig.1f

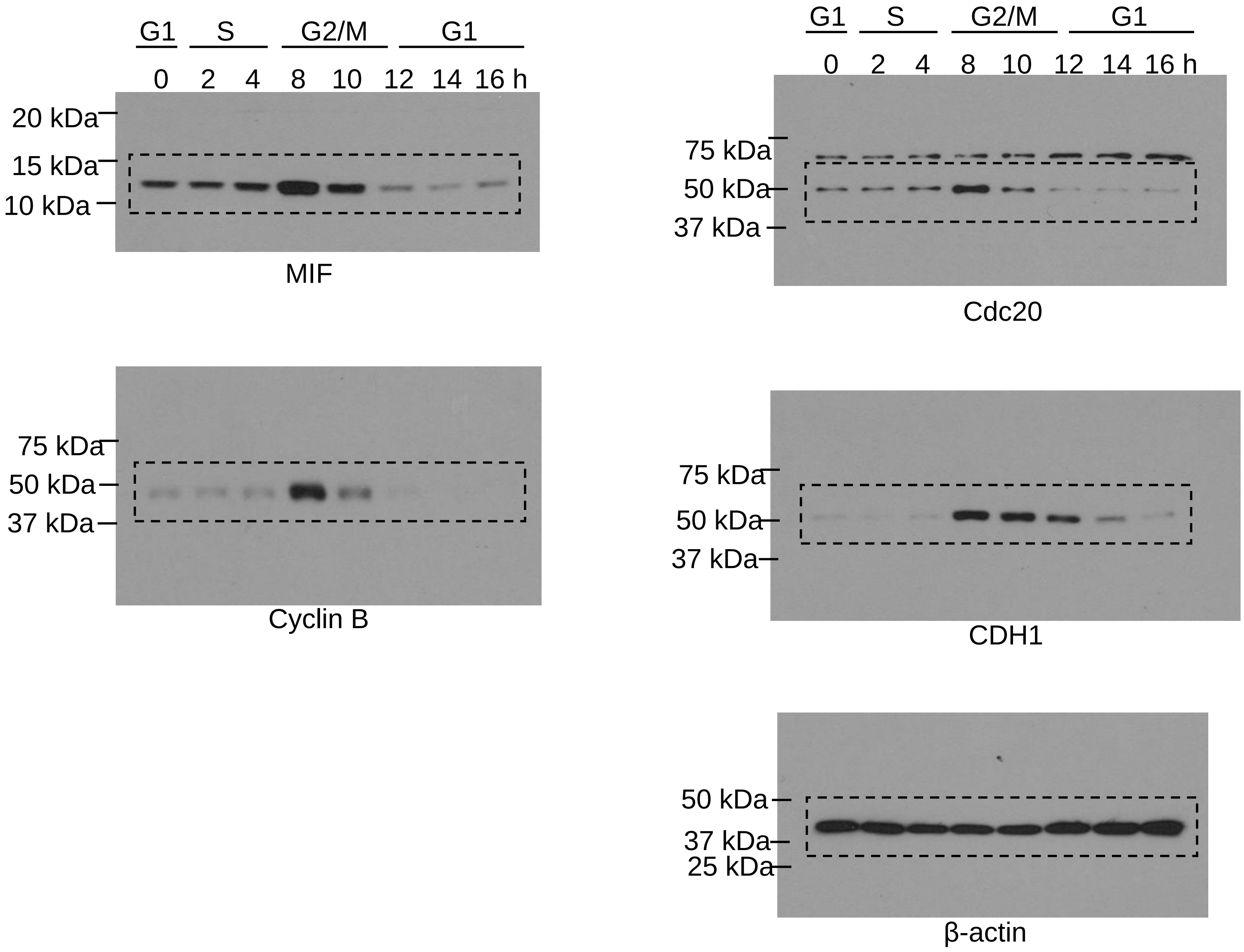

Source Data- western blots- Fig. 3b

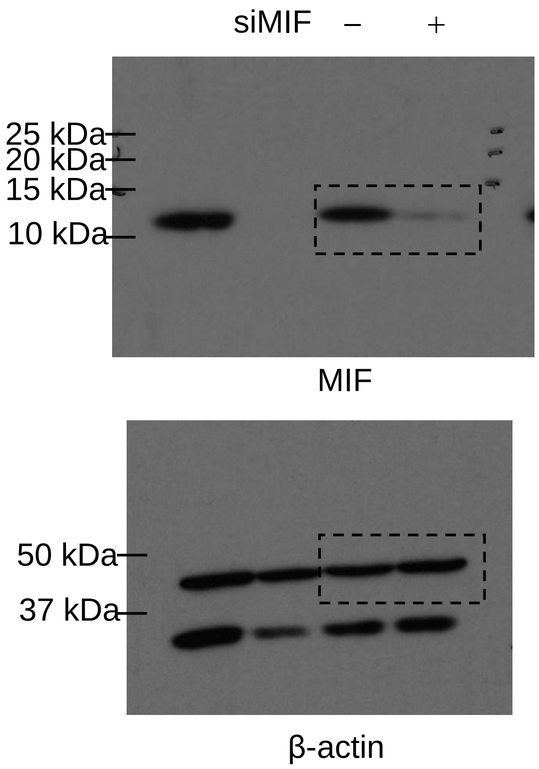

Source Data- western blots- Fig. 3e

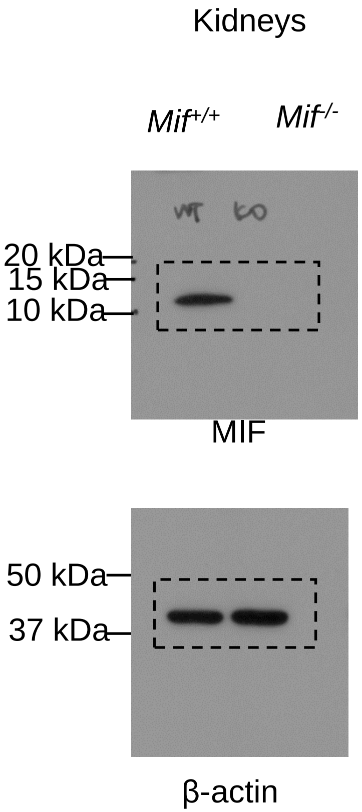

Source Data- western blots- Fig. 7d

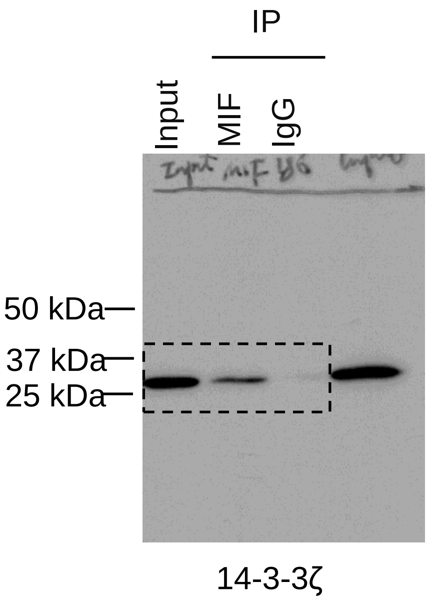

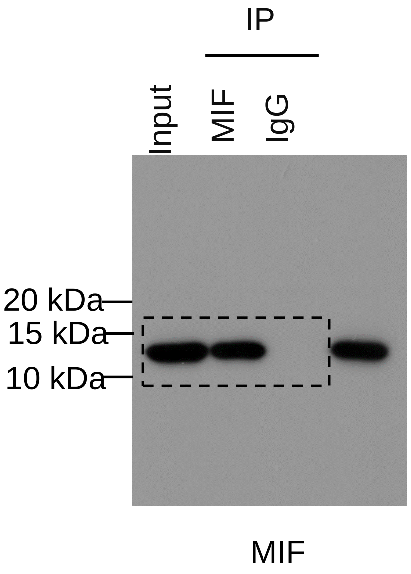

Source Data- western blots- Fig. 7e

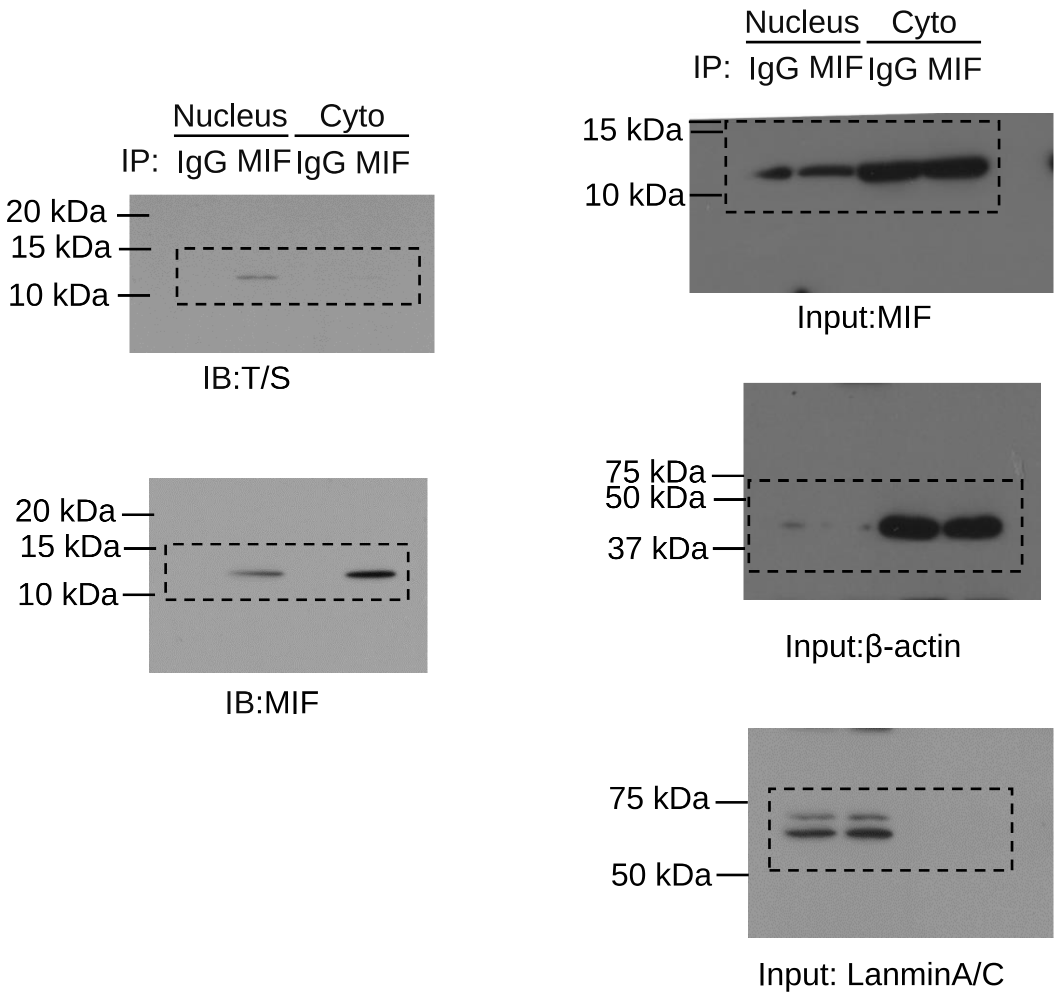

Source Data- western blots- Fig. 7f

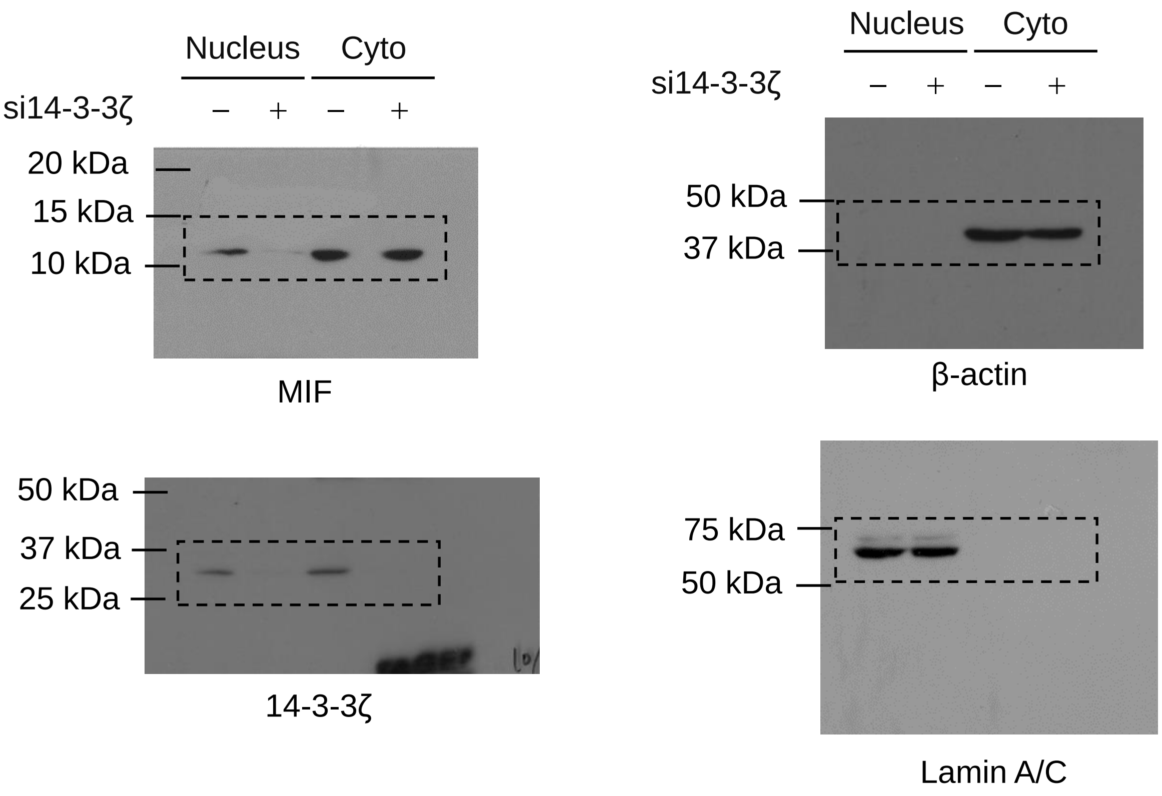

Source Data- western blots- Fig. 7i

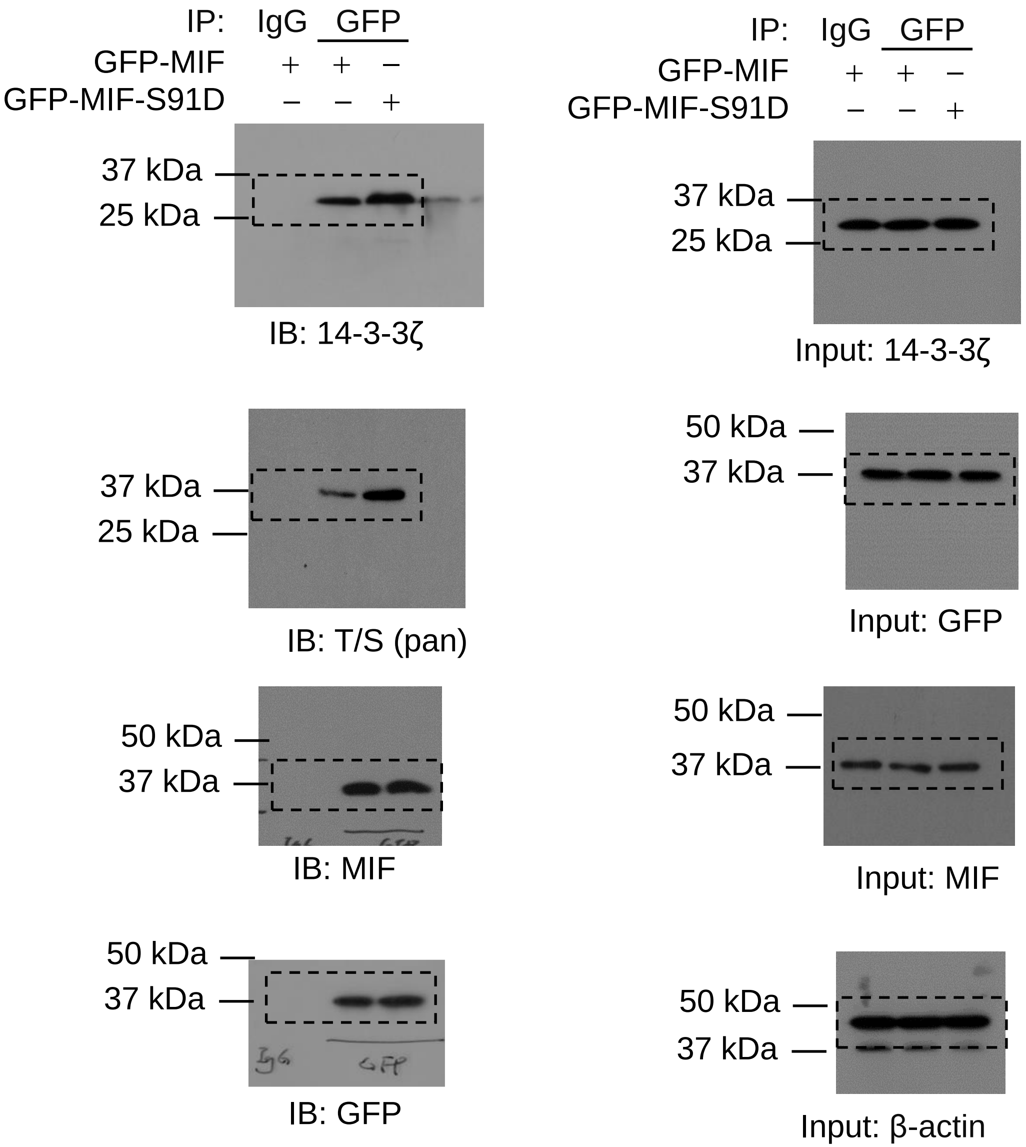

Source Data- western blots- Fig. 7j

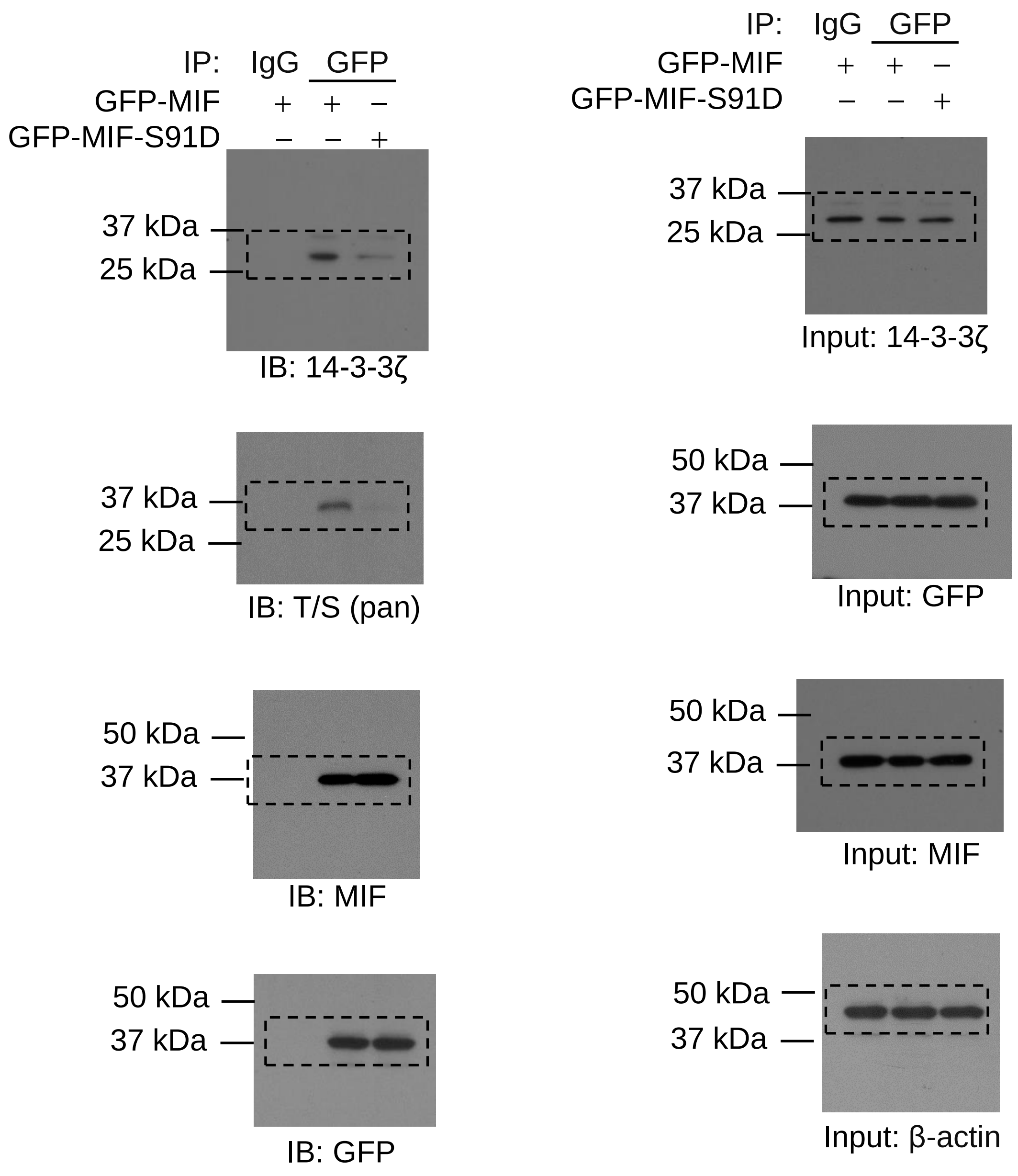

Source Data- western blots- Fig. 7k

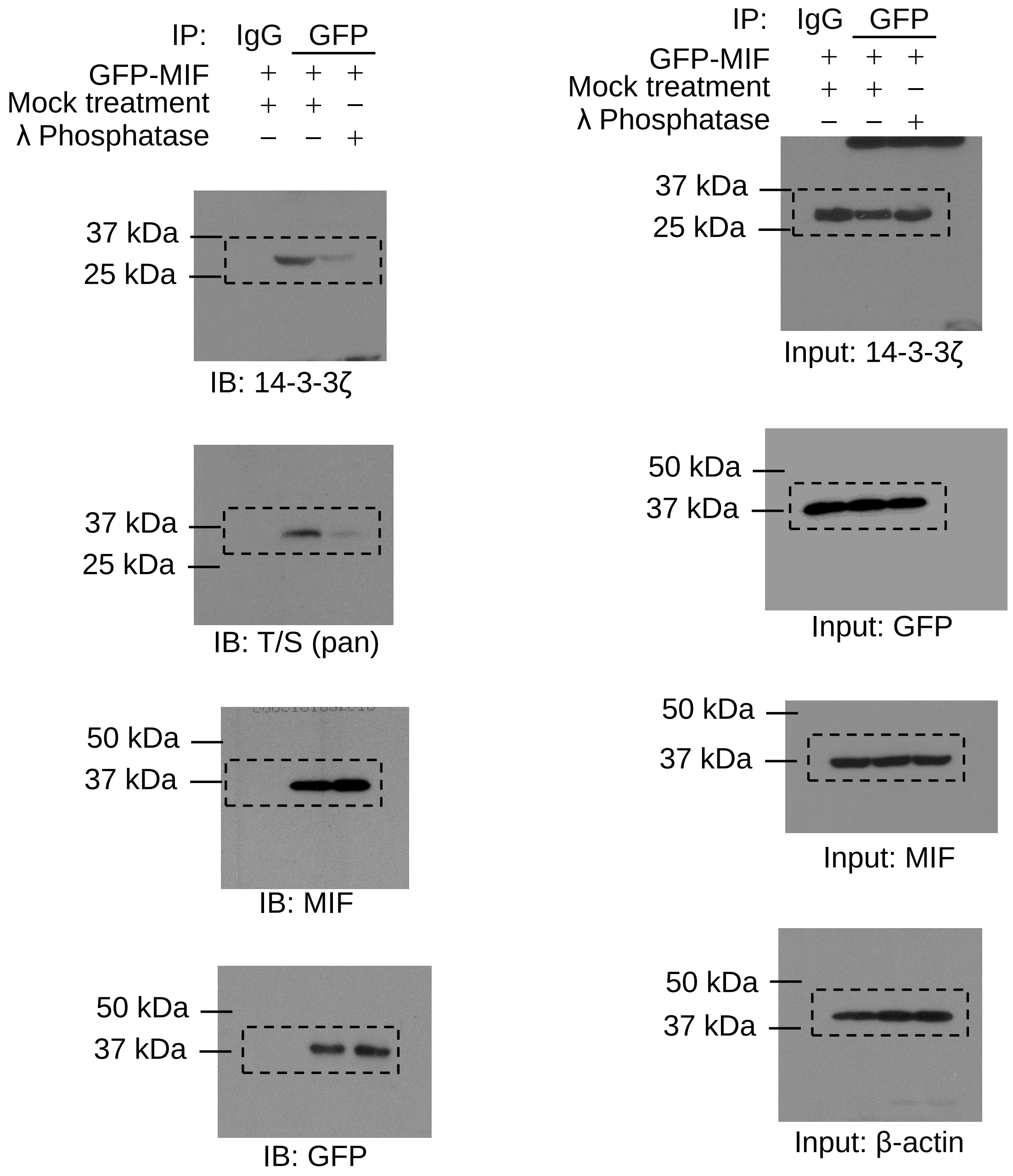

Source Data- western blots- Fig. 7n

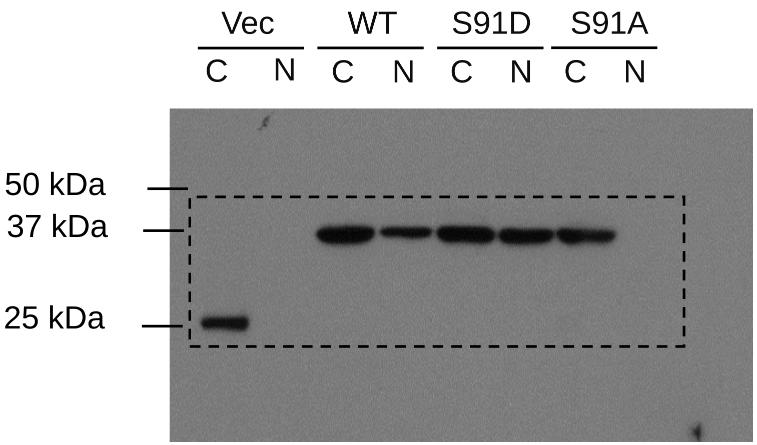

GFP

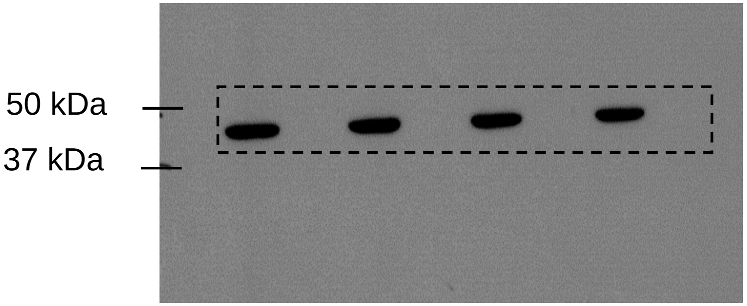

β-actin

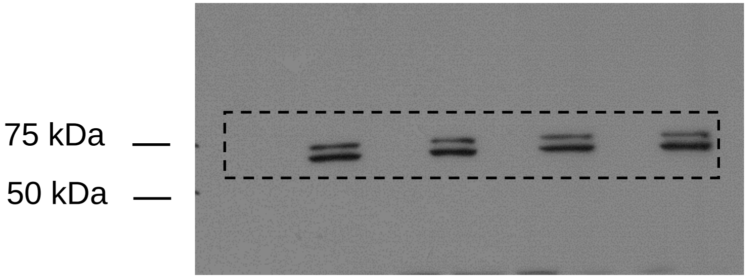

Lamin A/C

Source Data- western blots- Fig. 8a, b

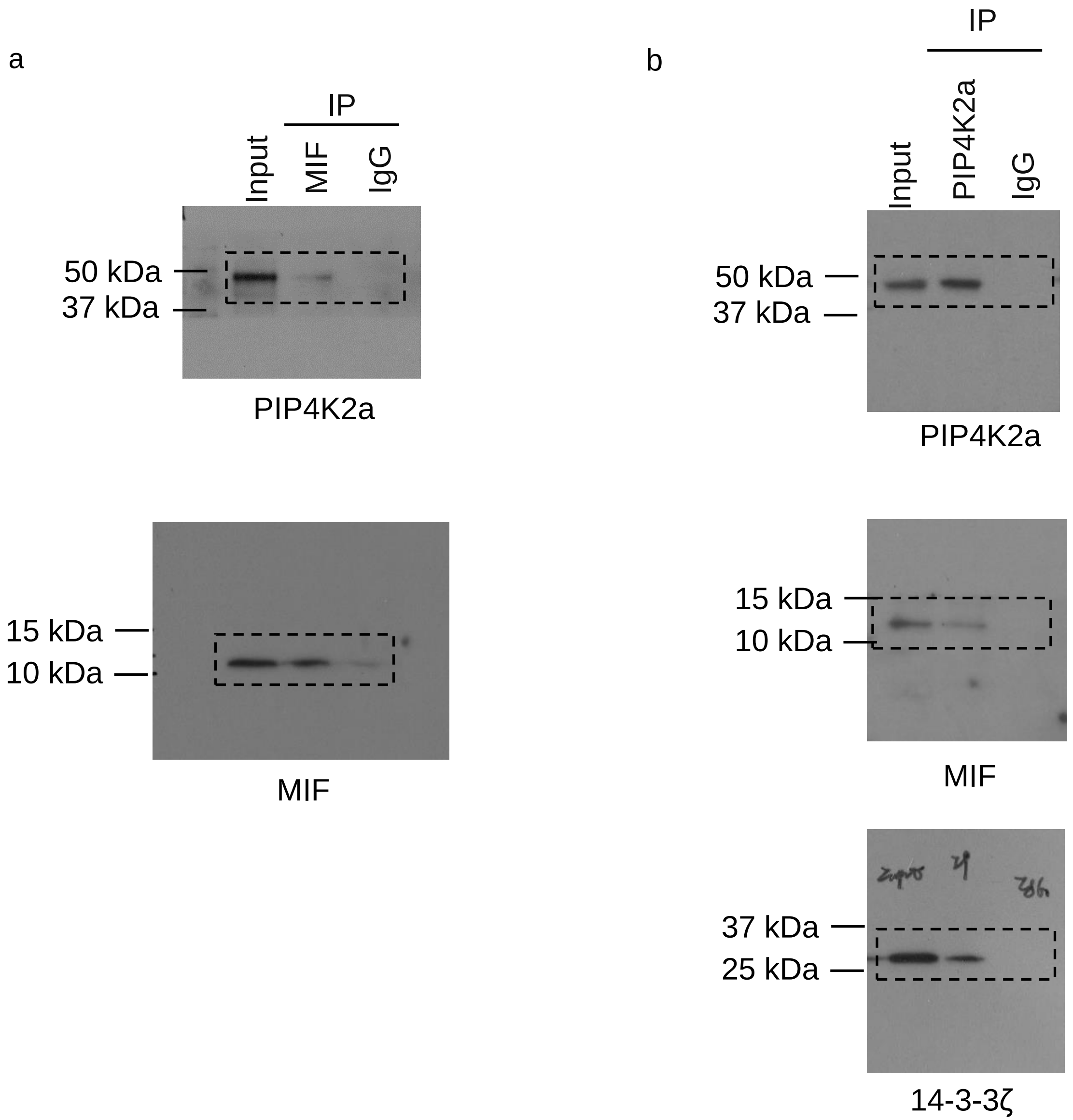

Source Data- western blots- Fig. 8c

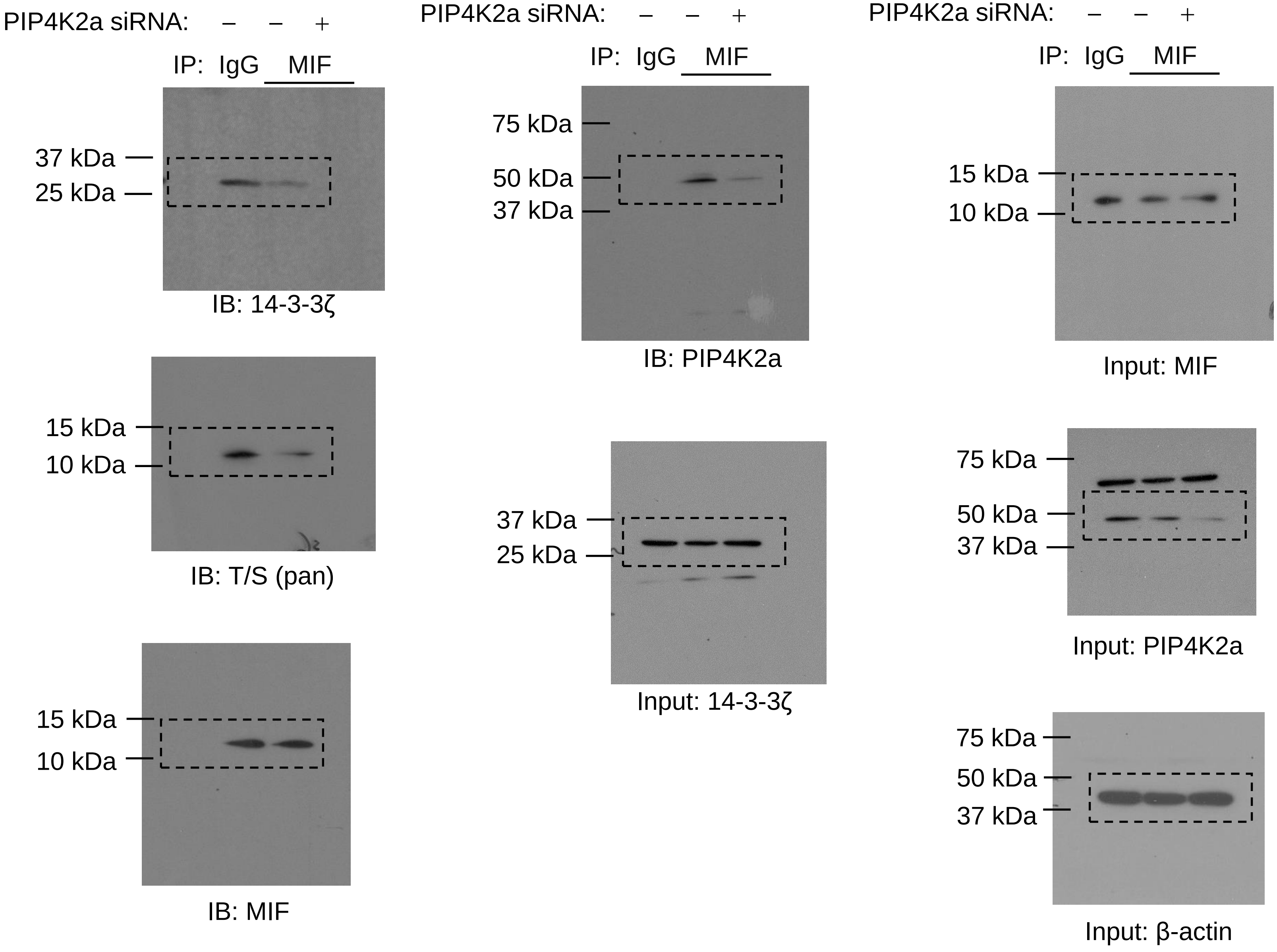

Source Data- western blots- Fig. 8d

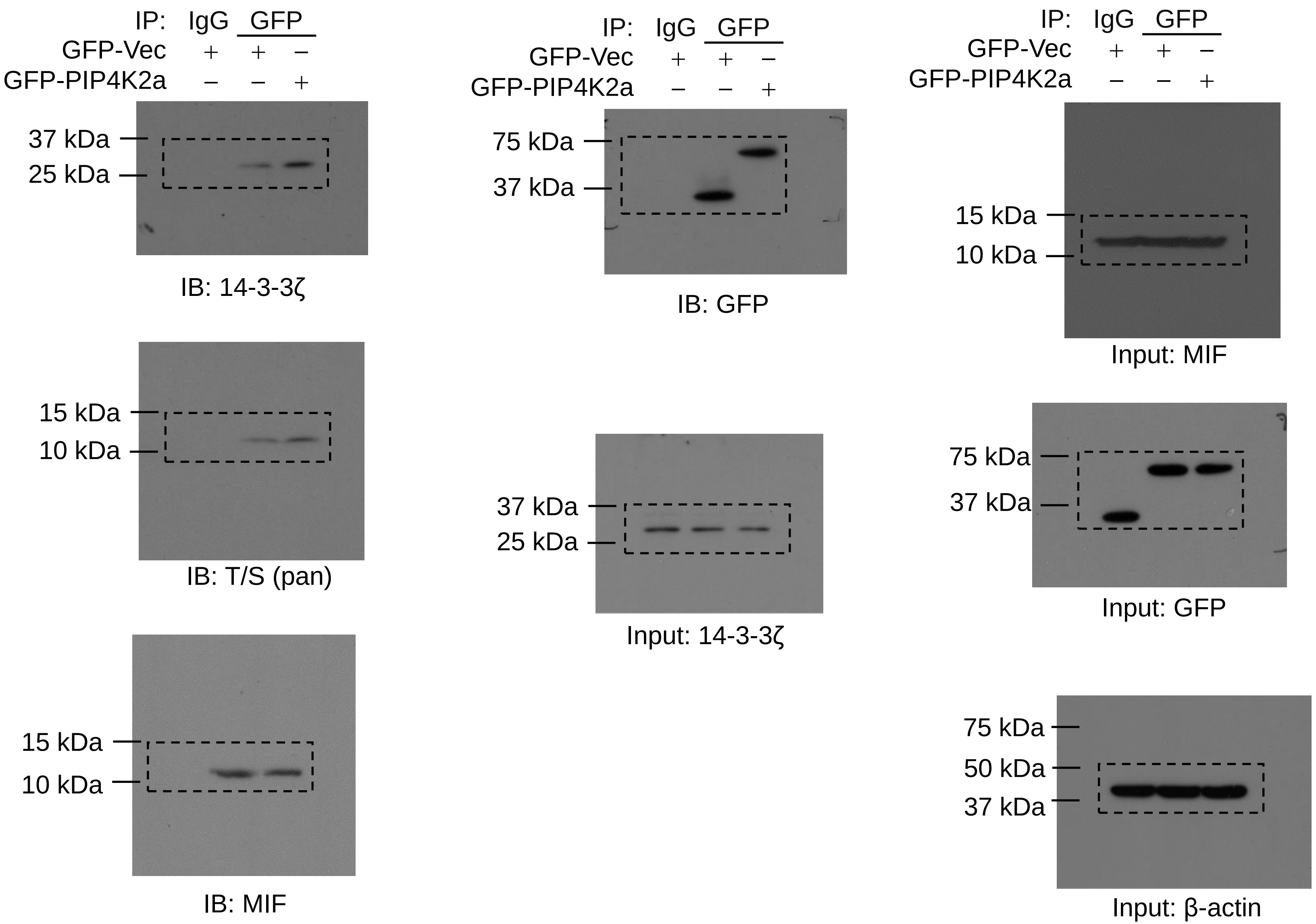

Source Data- western blots-Fig. S1c

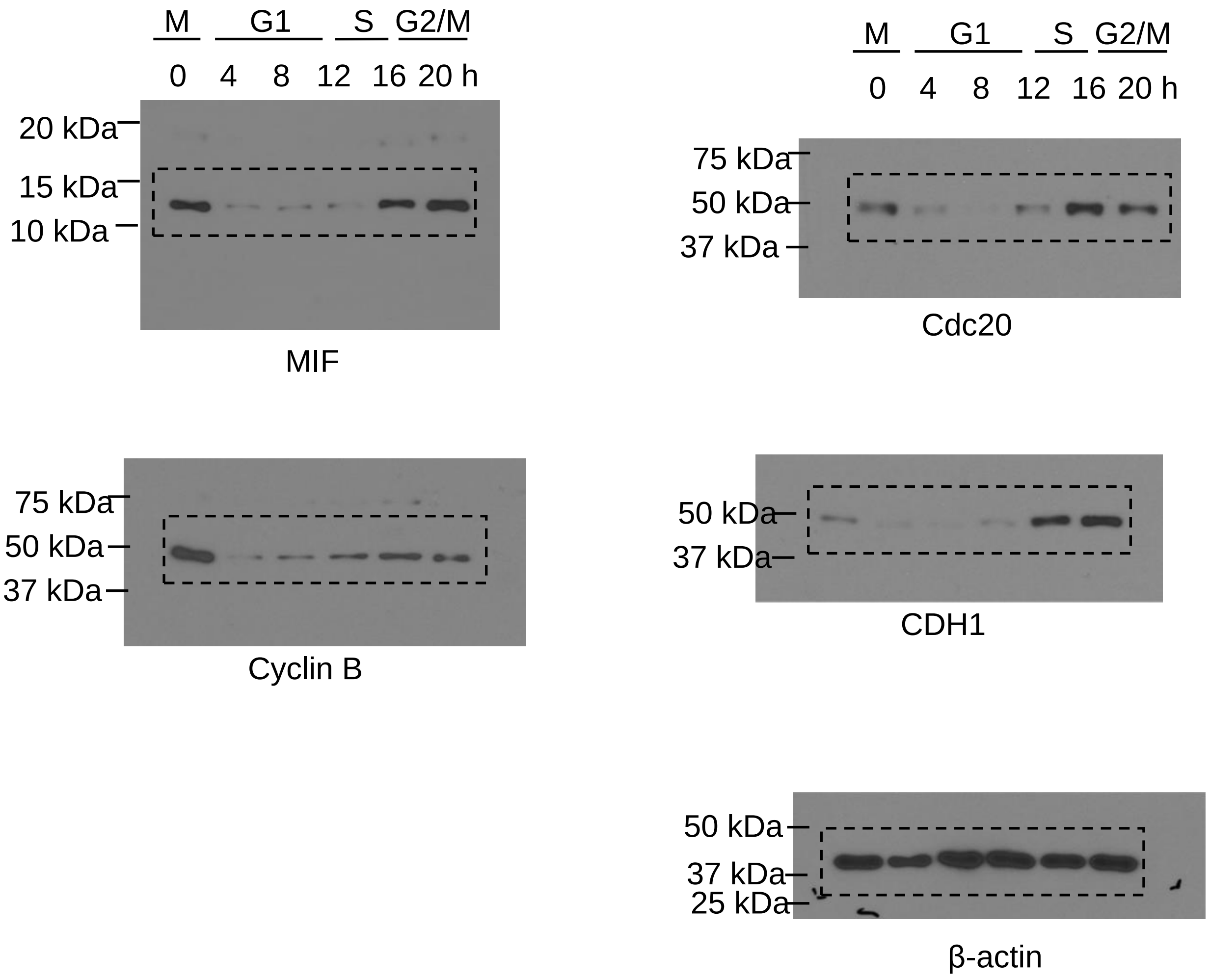

Source Data- western blots-Fig. S1d

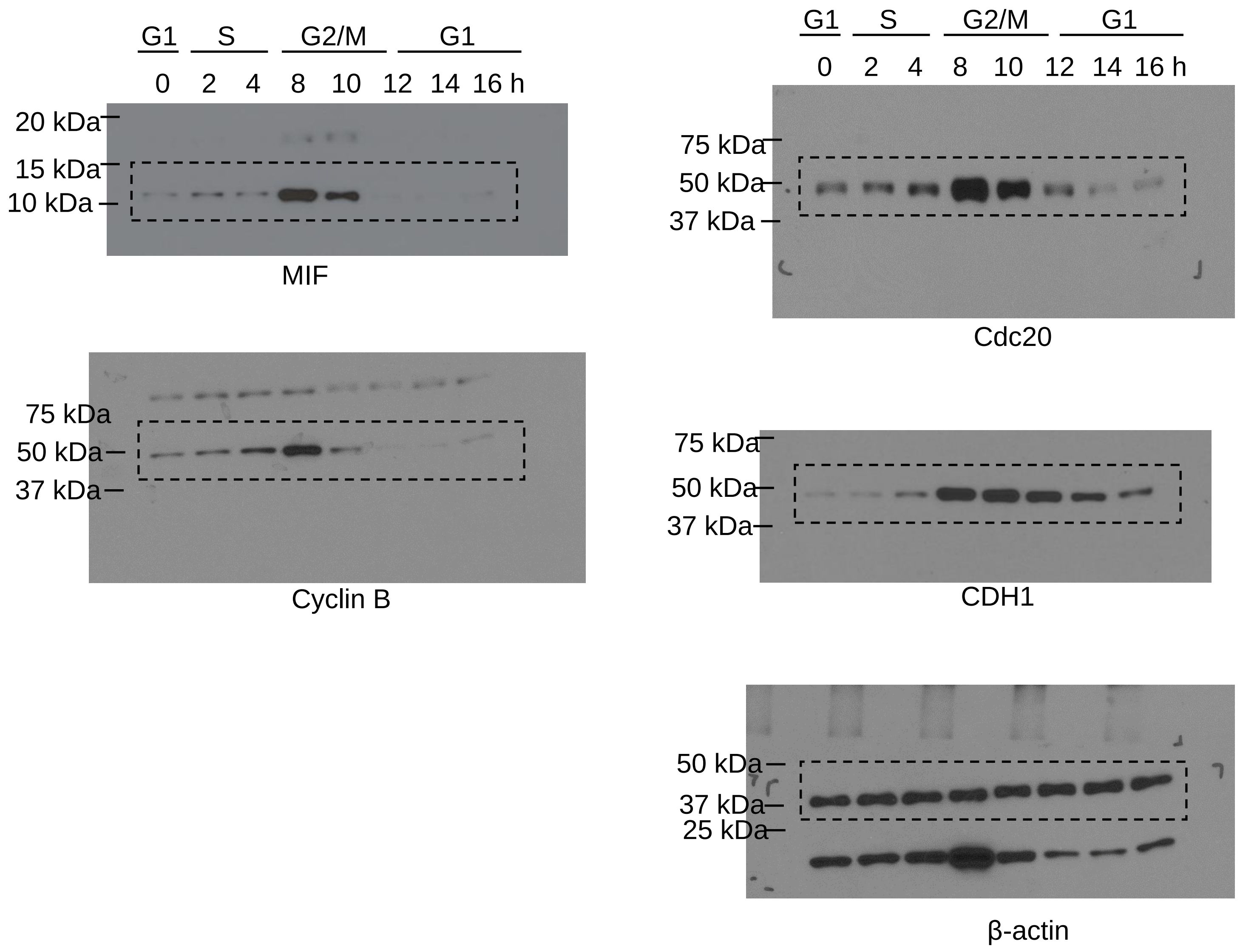

Source Data- western blots- Fig. S5b

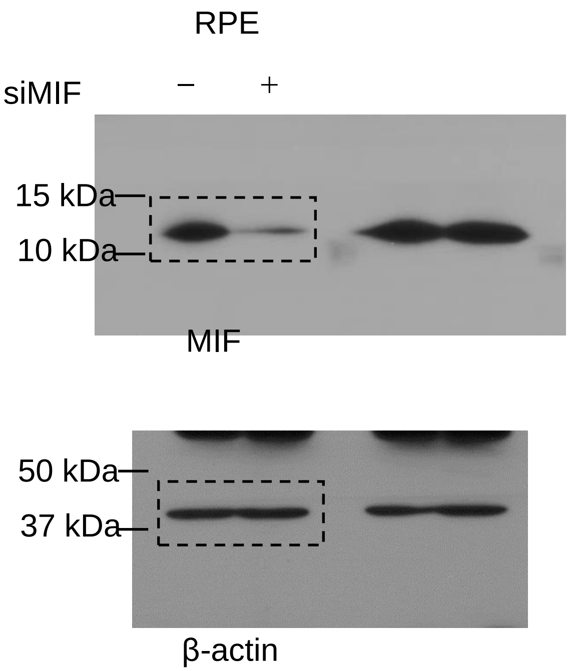

Source Data- western blots- Fig. S8c

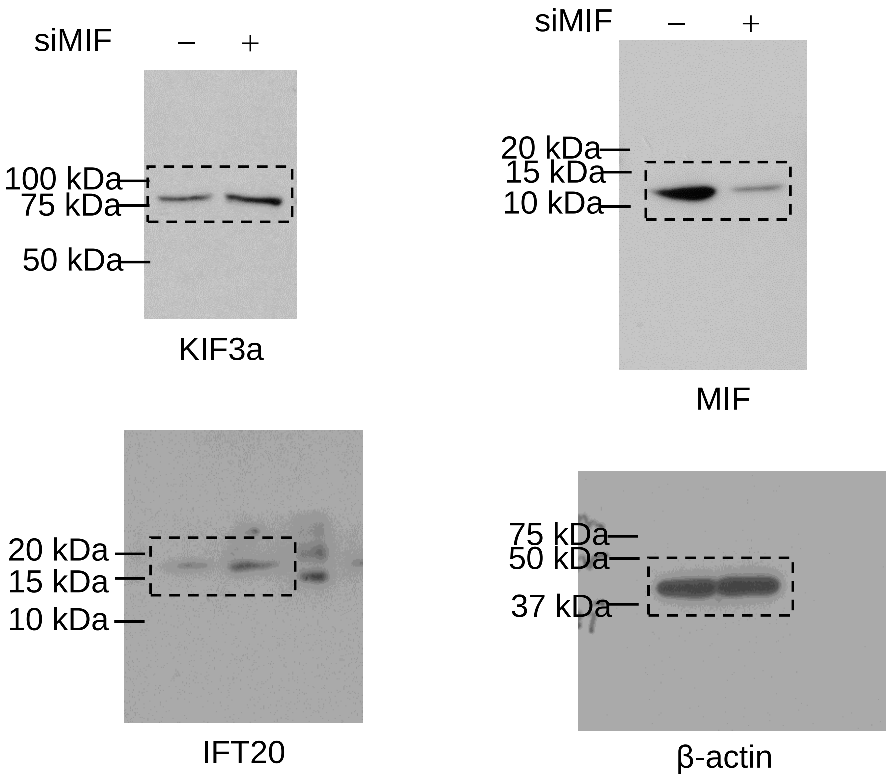

Source Data- western blots- Fig. S8f

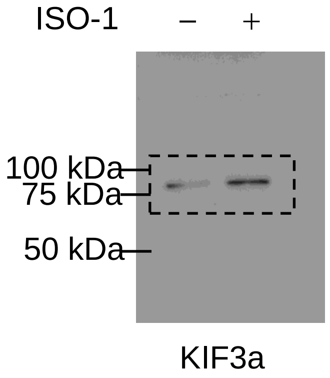

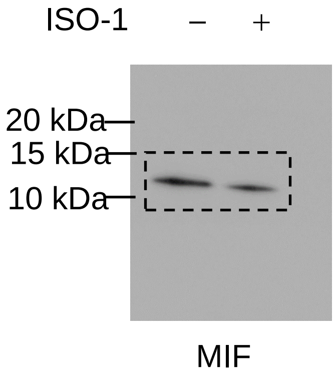

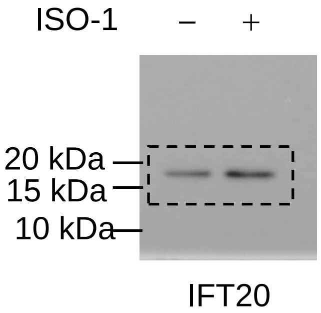

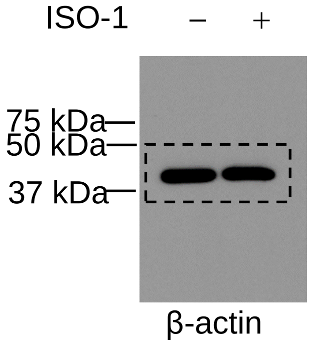

Supplement: Supplementary file 2 — Original Data File [file 41419_2023_6323_MOESM2_ESM.pdf]
